# An assessment of the ECETOC TRA Consumer tool performance as a screening level tool

**DOI:** 10.1038/s41370-022-00510-0

**Published:** 2023-01-21

**Authors:** Rosemary T. Zaleski, Tatsiana Dudzina, Detlef Keller, Chris Money, Hua Qian, Carlos Rodriguez, Frank Schnöder

**Affiliations:** 1 Lumina Consulting, L.L.C, Hillsborough, NJ USA; 2 ExxonMobil Biomedical Sciences, Inc., Machelen, Belgium; 3 Henkel AG & Co KGaA, Düsseldorf, Germany; 4 Cynara Consulting, Brockenhurst, UK; 5 ExxonMobil Biomedical Sciences, Inc., Annandale, NJ USA; 6 Procter and Gamble, Strombeek-Bever, Belgium; 7 DuPont de Nemours Deutschland GmbH, Neu-Isenburg, Germany

**Keywords:** Consumer exposure, ECETOC Targeted Risk Assessment tool (ECETOC TRA), Registration, Evaluation, Authorization, and Restriction of Chemicals (REACH), Dermal exposure, Oral exposure, Inhalation exposure

## Abstract

**Background:**

The European Centre for Ecotoxicology and Toxicology of Chemicals (ECETOC) Targeted Risk Assessment (TRA) Consumer tool was developed to fill in a methodology gap for a high throughput, screening level tool to support industry compliance with the European Union’s Registration, Evaluation, Authorization, and Restriction of Chemicals (REACH) regulation.

**Objective:**

To evaluate if the TRA Consumer tool has met its design of being a screening level tool (i.e., one which does not under-predict potential exposures).

**Methods:**

The TRA Consumer tool algorithms and defaults were reviewed and performance benchmarked vs. other consumer models and/or empirical data. Findings from existing reviews of the TRA consumer tool were also considered and addressed.

**Results:**

TRA predictions based on its default inputs exceeded measured exposures when available, typically by orders of magnitude, and were generally greater than or similar to those of other consumer exposure tools. For dermal exposure from articles, there was no evidence that a diffusivity approach would provide more appropriate exposure estimates than those of the TRA. When default values are refined using more specific data, the refined values must be considered holistically to reflect the situation being modeled as some parameters may be correlated.

**Significance:**

This is the first evaluation of the ECETOC TRA consumer tool in its entirety, considering algorithms, input defaults, and associated predictions for consumer products and articles. The evaluation confirmed its design as a screening level tool.

**Impact Statement:**

- The ECETOC TRA Consumer tool has been widely applied to generate exposure estimates to support chemical registrations under the EU REACH regulation. This evaluation supports the appropriateness of the TRA as a screening level exposure assessment tool. It also warrants additional measurements of consumer exposure, especially for article use scenarios, to aid the development of consumer exposure tools and chemical risk assessment.

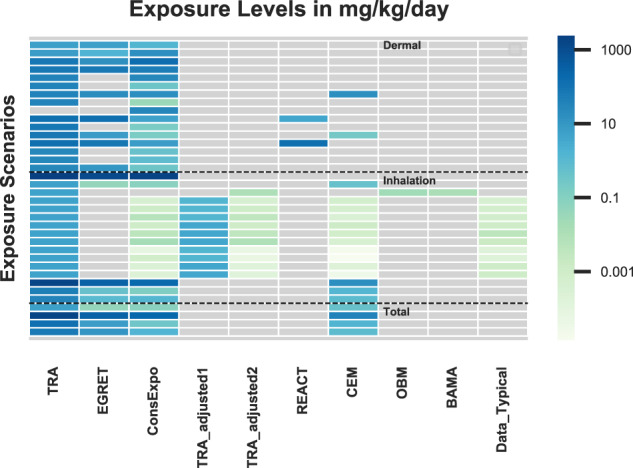

## Introduction

Since 2010, the ECETOC TRA Consumer tool has been a preferred screening level exposure tool for specifying conditions of safe use of consumer products under the REACH regulation. A full description of the TRA Consumer tool along with its historical development is provided in a number of ECETOC reports [1–5]. After an intensive and thorough review process by an ECHA-appointed ad-hoc expert group which included experts from ECETOC, RIVM, BfR, Danish EPA, ANSES, and ECHA^1^, the TRA Consumer tool was incorporated into ECHA’s Chesar exposure IT platform [6] in 2010. Ever since, the tool has been widely used for generating the consumer Chemical Safety Assessments necessary to support the REACH registrations of many substances.

The aim of ECETOC for the TRA consumer tool was to provide a pragmatic tool that could efficiently deliver exposure estimations for the many chemical substances and use scenarios required under the REACH regulation. Because of its intent to quickly screen many substances and scenarios as a first step, thereby removing some substance-scenario combinations from more detailed analysis but identifying others that should go on to assessment with higher tier tools, it was very important to ECETOC and all stakeholders that the tool design would not result in false negatives – i.e., not predict exposures lower than they would be in reality for the substance-scenario combination. Therefore, in the absence of specific use information, the scenario defaults were designed to be conservative representations of intended use conditions. Whether or not the degree of estimation would maximize the realism of the tool prediction was less of a concern as compared to the primary goal of avoiding false negatives (exposure underprediction).

Several studies have compared consumer exposure predictions by TRA and other models [7–12]. Generally, these papers have indicated the conservative nature of the TRA tool (i.e., TRA predictions exceed others) for most scenarios evaluated. One paper, however, has suggested that the TRA algorithm may not be conservative enough for dermal exposures via articles [12]. With these publications in mind, the goal here was to evaluate the conservative nature of the TRA tool in terms of its exposure calculation algorithms, default parameter values, and model predictions. The latter is particularly important [7]. Due to the interconnected nature of the algorithms and default parameter values, a predicted exposure could be more conservative even in a case where one or more default values were less conservative. In addition, parameters may also positively correlate with each other for some exposure scenarios (e.g., room volume and the amount of paint or tile glue used). Accordingly, it is important to consider the scenario holistically and the model prediction when trying to assess the conservative nature of a predictive tool.

This effort is not intended to be a statistical assessment of TRA tool performance (indeed one of the findings of this and other studies is that consumer exposure data needed for exposure model evaluation are generally sparse [6, 13]), but rather to consider the published findings of other tool users and also the results of recent studies specifically designed to address REACH exposure information requirements. Since the release of TRA, several downstream user sectors have published Specific Consumer Exposure Determinants (SCEDs) of values meant to be used to refine the TRA input parameters for specific scenarios when appropriate. In addition, a number of studies have been completed that are also relevant for evaluating the tool (EPHECT- Emissions, exposure patterns and health effects of consumer products in the EU, DRESS- DeRmal Exposure aSsessment Strategies, DustEx, SysDEA-Systematic analysis of Dermal Exposure to hazardous chemical Agents at the workplace). Specifically, it was important to assess if TRA predictions based upon built-in default values represent high end estimates of exposure (i.e., higher than measured or predicted values with other models for similar exposure scenario) that are still in line with reality.

## Materials and methods

This assessment focused on the current consumer TRA (v3.1) as run with default values and assuming a daily use^2^, using its stand-alone version. The TRA provides exposure estimates for both REACH Product Categories (PCs, which are generally liquid formulated products defined by their chemical constituents) and Article Categories (ACs, which are generally solid products defined by their shapes). Algorithms, default input values and exposure predictions were examined for PCs and ACs. A literature search was done on: a) the ECETOC TRA consumer tool and b) exposure data during consumer product use. Recent comprehensive reviews or analyses from other published studies were used when available. In addition to journal articles, data generated by regulatory agencies or other research studies designed to develop exposure data relevant for REACH application were considered.

### Algorithms (and scenario independent defaults)

Algorithms are presented and assessed relative to REACH Tier 1 algorithms [14] and other relevant guidance. In assessing the overall performance of an algorithm, scenario independent defaults are also considered as they impact the overall performance of the algorithm across scenarios. Published assessments of these aspects of the TRA are used and supplemented with additional information. Algorithms are examined by exposure route (inhalation, dermal, oral) and then by total exposure (sum of exposure across all routes per scenario).

### Scenario dependent defaults

The assessment of scenario dependent default values focuses on published assessments and data that have become available since version 2.0 of the TRA was released, as the defaults established in that version have not been changed. The consistency of default values is assessed and, when possible, the impact on TRA exposure predictions is also included.

### Comparison with other modeled or measured data

This evaluation focused on publications that have included model predictions using the consumer TRA and at least one other consumer model and/or measured data. In some cases, TRA predictions were developed for comparison to available modeled or measured data.

## Results

### PCs—Assessment of algorithms and scenario independent parameters

#### Inhalation

The tool structure is such that substances are assigned to one of 4 volatility bands based upon their vapor pressure (VP). For all bands, air concentrations are calculated based upon the following formula:

This equation is consistent with the instantaneous release, lowest tier algorithm in the RIVM ConsExpo model [15]. The full TRA equation provides exposure in mg/kg body weight/day units; event time, inhalation rate and body weight variables are omitted for air concentration. As compared to ECHA [14] inhalation equations for low tier assessments, the TRA algorithm considers a dilution fraction due to normal non-ventilated air flow between residential rooms and does not consider the respirable fraction for spray releases. TRA also includes a modifying factor (fraction released to air) for VP < 10 Pa and provides an upper bound air concentration based upon saturated vapor concentration (SVC). The SVC applies only to non-aerosols. Each parameter of the algorithm is assessed in detail in the Supplementary Information (SI). Here, we focus on the VP banding approach because it is the unique aspect of the TRA inhalation algorithm.

The VP band approach applies to non-aerosol products. For aerosols, the tool assumes that 100% of the substance in the product is instantaneously released to air. For non-aerosols, for the highest VP band it is assumed that 100% of the substance present in the product instantaneously volatilizes and becomes well-mixed within the room air. Thus, the algorithm prediction for this vapor pressure band will be the highest concentration possible based upon the input values of use amount and weight fraction. The assumption of complete mixing may underestimate air concentrations near the emission source in the first few minutes. The effect of this assumption, however, is greater with greater room size; for a 20 m^3^ room (the TRA default room size), the assumption was indicated to be reasonable [16].

**Fig. 1:**
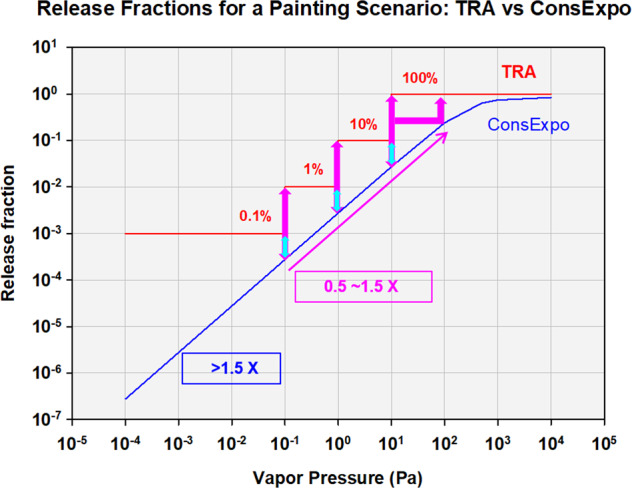
TRA Release fractions compared to ConsExpo release fractions. TRA release fractions in red, ConsExpo in blue. Inset boxes give differences in orders of magnitude.

At lower VP bands, for each order of magnitude decrease in VP, a factor of 10 reduction to the fraction released and therefore predicted air concentration is implemented. Modeling analysis was done to evaluate if these reductions provide conservative estimates for amount released for the scenario duration (Table 1, details in SI). The results for the painting scenario (Fig. 1) indicate that the TRA VP approach predicts release fractions 0.5–3.5 orders of magnitude higher than ConsExpo for the range examined; other scenarios gave similar results. Additional details (Table 1, SI) support an overall finding that the release fraction using the TRA VP band approach is conservative as compared with higher tier modeling and measured data. This is a result of the comparatively low cut-off of 10 Pa for the assumption of complete instantaneous release. Other authors have also indicated the conservative nature of the inhalation algorithm [7, 13]

**Table 1:** Data/Analyses to address assumptions of the TRA approach.

Analysis Type	Results				
**Aspect: Inhalation release factors based upon VP band**					
Modeling	TRA and ConsExpo evaporation mode predictions were compared, TRA more conservative (details in SI): • A linear association was observed between release fraction and VP in log scale, up to VP = 100 Pa • A release fraction of 0.5 would be conservative for vapor pressure bands up to 100 Pa, whereas the release fraction of 1 is currently applied at 10 Pa and higher. • For the lowest vapor pressure band, <0.1 Pa: ◦ At 0.1 Pa, TRA predictions are 0.5 orders of magnitude higher than ConsExpo with differences increasing as VP decreases- TRA estimates are 3.5 orders of magnitude greater than ConsExpo at the lowest VP examined				
Data Comparison	TRA predictions compared with measured data from two studies: • TRA release fraction 1–2 orders of magnitude higher than data (SI)				
**Aspect: Use of 0.01 cm thickness layer (TL) value for liquids dermal exposure**					
Data Comparison	USEPA 2011 [23]: Measured TLs of several liquids (varying in viscosity) on human skin exposed support the 0.01 value. Experimental TL values ranged from: • 0.005 to 0.011879 cm for total immersion scenarios without wiping, • 0.001–0.0042 cm for other scenarios without wiping, • 0.00001 to 0.0037 cm after partial or full wiping. • The highest TL value reported was for the immersion scenario (no wipe) of mineral oil and was 0.01187 cm. This was the only value reported that exceeded 0.01 cm (N=72).				
**Aspect: No specific pathway for dust ingestion (assumed conservatism in model would include potential exposure via dust ingestion)**					
Modeling	von Goetz (2016) [24] prepared two case studies comparing DustEx and TRA predictions. For each of the scenarios assessed, the contribution of dust ingestion was orders of magnitude lower than TRA total exposure predictions. For BPA/toy scenario results in ng/kg/day:				
		DustEx-Small Toy	DustEx-Large Toy	TRA-Toy(size not considered)	
	Tier 1:	35000	36000	Ingestion:	30000
	Tier 1.5:	0.015	0.015	Dermal:	1700000
	Dynamic:	0.000006	0.0004	Total:	1730000
	For DEHP/Plastic Objects scenario, results in mg/kg-BW/day:				
			DustEx	TRA	
	Small (non-accessible)		0.075	Inhalation = Total=2.6	
	Large (accessible)		0.75	Dermal 46, Inh 26, Total 72	
Data Comparison	Mitro et al. (2016 a,b) [25, 26] estimated exposures of 3–6 year old children via dust ingestion for 45 chemicals based upon a meta-analysis of US dust samples. Comparison to TRA inhalation and total exposure predictions using the lowest VP band (lowest predictions) is added here. Note that the dust ingestion exposure estimates of Mitro et al. were based upon total dust concentrations, which reflects multiple exposure sources as compared to the scenario-specific TRA estimates. Mitro: • Estimated exposures via dust ingestion for individual chemicals: < 0.001 mg/kg/day • [Equivalent to <0.003 mg/kg if adjusted to a 10 kg child body weight (TRA default) and a 100 mg dust intake] TRA, lowest VP band: • Inhalation predictions range from 0.0008–202 mg/kg/day (0.0008 is for subcategory tissue/paper, next lowest is 0.0036 subcategory glues hobby use) • Total exposures range from 0.07–1000 mg/kg/day At the VP band with the lowest inhalation prediction, TRA inhalation-only source-specific estimates were similar or up to 4 orders of magnitude higher than the reported estimates of dust exposure from all sources, and TRA total source-specific exposures 1–5 orders of magnitude higher.

#### Dermal route

The dermal algorithm is:

The dermal transfer factor (TF) in the TRA represents the fraction of the substance in the thickness layer (TL) in contact with the skin that is transferred to the skin [3]. The default TF remains at 1 for all scenarios within the TRA. The TF does not refer to or account for the fraction of material that might be subsequently absorbed through the skin into systemic circulation.

The algorithm is consistent with that provided in the EU Technical Guidance Document (TGD) [17], and ECHA [14], although frequency of use and the potential to consider a transfer factor is included. In TRA default mode, both factors are set to 1 and so have no impact on the prediction. The TRA algorithm assumes that there is a uniform TL on skin across the whole contact area and that the total amount of substance in this uniform layer is available for absorption.

For PCs, a value of 0.01 cm is used as the TL for all scenarios with the exception of 0.001 for air care, continuous action, solid (the latter will be addressed with articles since it is a solid). Reduced thickness of a (uniform) layer of 0.001 cm for some ACs has been set in TRA to account for the reduced mobility of substances in the article matrix and is applied here to the solid air freshener. This approach has been challenged for articles and will be discussed in detail in that section; here we provide the basis for the approach for liquid products. A default TL value of 0.01 cm has been used for years for liquids and is identical to the TGD default TL of 0.01 cm for non-solid media in contact with the skin [14, 17–22]. Data on liquid thickness layer on skin are summarized in Table 1 and support that 0.01 cm is a conservative value [23]. The TL approach implies a dermal load of 10 mg/cm^2^ across the entire exposed skin surface area, which is close to the maximum that may be reasonably assumed (ca. 12–14 mg/cm^2^) according to the EU TGD [17].

Additional discussion of the dermal algorithm can be found in SI Section 4.

#### Oral route

The ingestion algorithm is:

The algorithm is comparable with that in the EU TGD [17] and ECHA [14] for initial tier oral assessments, with the addition of the possibility to consider a transfer factor (TF). As the TRA default TF = 1 for all scenarios, this capability does not impact the conservative nature of default assessments (SI).

The TRA does not include a separate dust ingestion pathway for non-intentional mouthing exposure. This pathway would primarily apply to semi-Volatile Organic Compounds (SVOCs) and non-VOCs, as VOCs would partition to air rather than dust. The TRA assumes that at least 0.1 % of any compound (lowest VP band) evaporates immediately and is inhaled in the standard room with standard ventilation. In addition, depending upon the scenario, oral and/or dermal exposures may occur as well, in which exposure is calculated based upon 100% of the substance present in the ingested or dermal contact mass. Total direct exposure is intended to exceed the potential exposure contribution via dust ingestion.

Comparison of TRA predictions with those of the recently developed DustEx dust specific model and exposure estimates based upon measured data (Table 1) [24–26] support that, if used with default assumptions, TRA predictions should cover exposure via the pathway of dust ingestion as well. If the TRA is run with refined assumptions in a case where dust ingestion may occur, then the user should assess if there is a need to evaluate dust exposure independently (for example via the DustEx framework) [24]. A recent analysis [27] indicates that dust-mediated transfer is most notable for substances with intermediate octanol-air partition coefficients (${{{{{\rm{k}}}}}}_{{{{{\rm{OA}}}}}}{10}^{6}-{10}^{10}$) where indoor partitioning is mediated by air.

#### PCs—total exposure

Within the TRA, each route equation operates independently, i.e., even if the inhalation algorithm indicates 100% of the substance is present in air, the dermal (and oral) algorithms are unchanged and provide additional exposure predictions for substance present on skin or ingested when relevant. Mass balance is exceeded in these cases, which will result in additional conservativeness in the tool.

### PCs—scenario dependent parameterization

Default values for TRA scenario dependent parameters (Table SI-6.1) were compared with alternate values found within published Specific Consumer Exposure Determinants (SCEDS) or other public sources of these data (Table SI-6.2). Comparison of values for individual parameters, however, is of limited utility as it is the combination of parameters and the model algorithm that determine the relative conservatism in the exposure assessment. For example, in the CONCAWE SCEDs the default weight fraction has been raised to 1 in all cases, yet the exposure estimates are lower than those based upon the TRA defaults due to refinements in other values (Fig. SI-6.1) [28]. Overall, most SCEDS or other sources of defaults were similar or less conservative than those in the TRA. In some cases, data were available for only one parameter and its impact on the scenario as a whole could not be assessed (for example, paint use amount was provided but without indication of room size or exposure duration [29]). For the SCEDS, which provide complete scenario information, predicted total exposures were lower than those of the TRA with the exception of CEPE scenarios in which exposure time was increased or, in the case of spray painting, a dermal route was added in the SCED [30]. The significance of the dermal route for this scenario will be assessed in the next section.

### PCs—comparison to modeled predictions or measured data

**Fig. 2:**
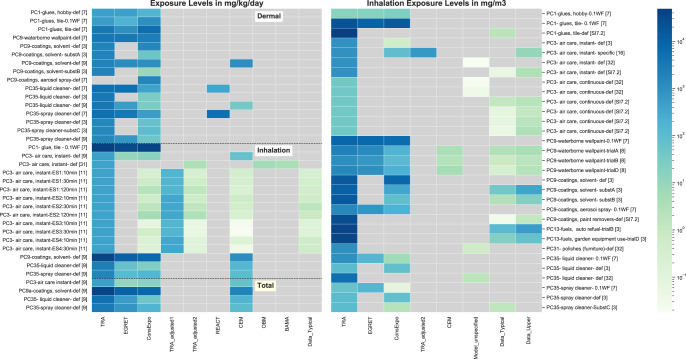
PCs: Comparison of TRA exposure predictions with predictions of other models and available data. Note log scale of coloring; the same scale applies to both mg/kg.day and mg/m^3^. Gray color indicates not assessed (no information). Vertical axis provides product category, scenario basis (def = default or spec=specific modifications; if only weight fraction was modified the modified value is indicated as ‘x’WF) and source (reference number, table number, or supplemental information section). Horizontal axis provides the name of the model used to generate the prediction or if the exposure values are data-based (if so, whether a typical or upper bound value). Additional details for all are found in Table 2 and/or supporting information.

Table 2 and Fig. 2 (and SI) present results from various studies that have compared TRA predictions with other models and/or measured data [3, 7–9, 11, 16, 31, 32]. In some cases, the TRA was run with defaults; when measured data were available it was generally modified to match the exposure scenario conditions.

**Table 2:** Summary of benchmarking for TRA predictions with models and/or data.

Reference	Benchmark	Scenarios	Results		
Oltmanns et al. [7]	TRA EGRET REACT ConsExpo	PC1–1 bottled glue PC1–2 tile glue PC9a-1 waterborne wall paint PC9a-3 spray can PC35–2 all purpose cleaner PC35–3 glass spray cleaner	**Long-term exposure:** TRA always highest, sometime by orders of magnitude **Event exposure:** *Inhalation (mg/m*^*3*^)(Fig. SI-7.1): Oltmanns adjusted the scenario weight fraction to 0.1 for all. TRA highest for all scenarios except: • PC1–1-similar order of magnitude as other models. For PC1–1 TRA used a default of 9 g whereas ConsExpo used 10g, Oltmanns deems both to be reasonable • PC35–3- EGRET > TRA > ConsExpo. For PC35–3, EGRET assumed a shorter exposure duration leading to a higher air concentration (less ventilation dilution) for the shorter period. • Note, TRA predictions with default weight fraction would exceed all others. *Dermal*: TRA highest dermal estimates for all except for: • PC1–2- ConsExpo assumed a dermal contact rate of 30 mg/min over the entire application period of 6 hours based upon data for painting, and it is noted that this may not be representative for tile glue use. For PC1–2 *total* exposure: TRA highest • TRA did not include a dermal estimate for PC9a-3 whereas ConsExpo did.		
ECETOC [3]	ConsExpo TRA Data	Models: PC3 air care instant PC9a coating solvent PC13 auto refuel PC13 garden equipment refuel PC35 liquid cleaner PC35 spray cleaner Data: PC9a, 13, 35-spray	See Figs. SI-7.2, S-I7.3 **TRA vs ConsExpo:** Dermal – TRA highest Inhalation (mg/m^3^)- TRA highest **TRA vs. Measured** TRA orders of magnitude higher		
Cowan Ellsberry et al. [9]	TRA ConsExpo CEM	PC3 air care instant PC8a coatings solvent rich PC35 liquid cleaner PC35 trigger spray cleaner	See Figs. SI 7.4–7.6 Inhalation (in mg/kg/day): TRA highest Dermal (mg/kg/day): TRA highest		
Feld-Cook et al. [8]	TRA EGRET Well-Mixed-Box ConsExpo E-FAST ART (median) Data	PC9 painting, latex	See Fig. SI 7.7 Inhalation (mg/m^3^): TRA predictions highest TRA exceeded measured concentrations by orders of magnitude		
Steiling et al. [31]	One Box Model, BAMA	PC3 instant action air freshener, did not include TRA (added in here)	See Fig. SI 7.8 TRA default predictions orders of magnitude higher. TRA adjusted to Steiling scenario gave similar results (both used same equations)		
Park et al. [11]	TRA CEM SprayExpo ConsExpoWeb ConsExpoNano Data	PC3 air care instant action aerosol spray, data for mass concentration of nanoaerosols	See Fig. SI-7.9 TRA default for fraction of substance released to air is 100%, Park predictions were based upon refinements including reducing the release fraction to 1–6% across scenarios. Parks SI included TRA default predictions partially adjusted: these were orders of magnitude higher than other model estimates and aerosol mass monitoring and subsequently refined Models adjusted to the scenario conditions showed variability in model relative ranking. See Delmaar 2020 for further information.		
Delmaar and Meesters [16]	ConsExpo Data	PC35 data for all purpose spray cleaner and also reran ConsExpo for the Park(2018) 0-ACH scenario	See Figs. SI 7.10–7.11 TRA model runs developed for comparison using only defaults and also adjusted to match scenario conditions. The TRA predictions exceeded both the measured and ConsExpo modeled air concentrations. Delmar also reran ConsExpo models for the Park (2018) 0 ACH scenarios using a weight fraction range of 0.01 – 0.1, as the 0.1 value was based upon the MSDS concentration and likely represented an upper bound. When the Park parameter values are incorporated in the TRA, the TRA event averages are an order of magnitude greater than the ConsExpo modeled peak or the measured event peak concentration		
Dimitroulopoulou et al. [32]	Modeled exposure for several agents in household products based upon the Emissions Exposure Patterns and Health Effects of Consumer Products in the EU (EPHECT) data on usage and also measured emissions.TRA comparison added here.	PC3 air care instant PC3 air care continuous action PC35 liquid cleaner PC35 spray cleaner	TRA predictions are generally orders of magnitude greater. The weight fraction present in the products is not provided, but included here are the maximum 30 min air concentration of any of the substances looked at by product category for 0.5 ACH conditions (or the next closest ACH > 0.1), and also the maximum 30 min air concentration resulting from all uses within the household (aggregate exposure). Estimated Air concentrations in µg/m^3^ by product type		
			Product	EPHECT	TRA
			Liquid cleaner	1715	5220000
			Furniture polish spray	64	993000
			Air care:		
			- instant action, aerosol spray	18	870000
			- continuous action, solid and liquid	22	43000
			- continuous action, solid and liquid	38	43000
			All Products Combined:	1716	
Additional comparisons	Measured air concentration data	floor adhesive, air freshener-gel, liquid, electric diffusor, spray, paint remover	See Table SI 7.2 for full references and details. TRA predictions generally orders of magnitude greater Air Concentrations in mg/m^3^		
				Measured TotalVOC	Prediction TRA
			Floor adhesive (low VOC)	1.84	49500
			Air freshener-gel	0.08–2.2	43.1
			Air freshener- liquid	0.08–2.0	43.1
			Air freshener-spray	0.06–7.3	870
			Paint remover	Single substance: 0.03–1.2	Using substance VP and MW: 265000
Marquart [33]	Occupational Data	Measured data for dermal exposure to hands (820 cm^2^)	See Table SI 7.3 for full reference and details. Several scenarios relevant to consumer TRA summarized, provide ranges of 2–7 mg/kg per scenario for spray painting, spreading floor glue, and painting with a brush.Specifically, for the Marquart scenarios and the closest TRA scenarios compared, Dermal exposure for hands (mg/kg body weight):		
			Marquart		TRA Prediction
			Car body spraying with liquid paint: 1.7		Spray paint: none
			Spreading parquet lacquer with a comb: 2		Floor adhesive: 42.9
			Painting window frames with a brush: 6.7		Painting with brush: 35.7
Franken et al. [34]	Systematic analysis of dermal exposure (SysDEA) study designed to assess dermal measurement techniques for occupational scenarios		Spray exposures were for 20 minutes. Spraying hand exposures normalized to a 0.2% weight fraction ranged from: • 0.05–1 mg per event and total body exposure 0.311–5.386 mg. • Geometric means for total body exposures were 0.5–2.7 mg. • If total body geometric means were adjusted to a 0.5 weight fraction as per TRA spray scenario, they would be 126–673 mg (or 2–11 mg/kg for 60 kg body weight). The range for hands would be 125–250 mg or 0.2–4.2 mg/kg body weight. Most of dermal exposure found on hands for pouring, rolling, and immersion/dipping. For spraying exposure was more uniformly distributed across body.		
Utilizing above studies, analysis to assess the impact of not including a dermal route for PC spray paint	Modeling, Data	PC9a spray paint	See SI8 for details. For spray paint scenario, used modeled estimates from Oltmanns et al. [7] for 0.1 weight fraction, measured dermal two-hands exposure data from SYSDEA [34] adjusted to 0.1 weight fraction. Exposures in mg/kg/day: • TRA: 10.3 Inhalation = Total • ConsExpo: 1.4 Inhalation + 30 Dermal= 31.4 Total • SYSDEA Dermal adjusted to 0.1 weight fraction: 0.8 • ConsExpo 1.4 Inh + SysDEA 0.8 Derm = 2.2 Total • TRA all defaults, 0.5 weight fraction: Inh = Total =51.4 For 0.1 weight fraction: ConsExpo Inh + SysDEA Derm (2.4) < TRA Inh (10.4) < ConsExpo Inh + Derm (31.4) • TRA inhalation prediction is lower but similar order of magnitude as ConsExpo Inh+Derm; • TRA inhalation prediction exceeds total exposure when dermal is based upon SYSDEA data; • TRA default prediction for scenario exceeds weight fraction adjusted predictions.		

Information underpins Fig. 2.

Overall, these analyses do not cover all PC codes, but the general trend to provide the most conservative results across publications and models and measured data support the finding that the TRA is an appropriate Tier 1 tool. In the few cases where other tools provided greater route-specific predictions, generally they were within a factor of 2.5 of those of the TRA, were also intended to be conservative predictions and/or there was uncertainty in the defaults applied in other tools. In all cases with measured data, the TRA default predictions far exceeded measurements. In the one case where TRA inputs refined by the authors provided predictions less than measured data [11], information was insufficient to address all of the refinements for appropriateness with TRA use.

The comparison to measured data was, however, limited to air concentrations. No monitoring data for consumer dermal or ingestion routes were identified for PCs and ACs included in the TRA. To address this gap, recent dermal occupational data were evaluated for relevance (Tables 2, SI) [33, 34]. These occupational data support that the tile glue and brush painting dermal estimates in TRA are appropriate. They also suggest that for completeness, particularly in cases where the dermal route is of specific interest, it may be useful to consider a dermal route i.e., for spray paint. Note, however, that in general the TRA is not meant to provide route-specific values but overall systemic exposures (RCRs are added across routes). The expectation would be that when route specific exposures are of interest, for example because of acute effects, more refined tools may be available to address those situations.

A more detailed analysis of the impact of TRA not including a dermal route for the spray paint exposure scenario is also provided in Tables 2. Adding a dermal exposure estimate based upon the SysDEA data to the ConsExpo inhalation estimate for a 0.1 weight fraction (based upon the scenario in Oltmanns et al. 2015) provides a total exposure lower than the TRA inhalation prediction for a 0.1 weight fraction.

### ACs—assessment of algorithms and scenario independent parameters

Inhalation, oral and dermal algorithms remain the same for articles.

#### Inhalation route

In general, the inhalation algorithm will be more conservative when applied to articles than products. Whereas liquid products are generally applied to surfaces in thin layers, substances present within articles need to diffuse to the article surface to become airborne. Thus, the TRA assumption of instantaneous release at the start of an exposure scenario is a further departure from reality for articles as compared to liquids.

As many substances in articles will be non-VOCs or SVOCs, it is useful to consider if inhalation estimates for the lowest VP band (<0.01 Pa, 0.001 of total amount in product instantaneously released) are conservative. The modeling analysis provided earlier indicates that it is, and that as VP is reduced further beyond the 0.1 Pa cutoff for the lowest VP band, the conservatism increases (Fig. 1).

#### Oral route

The assessment presented earlier applies to articles as well. The algorithm’s assumption that 100% of the amount placed in the mouth is ingested is more conservative for articles than products, as it assumes total ingestion or migration out of the solid item placed in the mouth. The overall level of conservatism in the prediction will depend upon the scenario dependent values of amount ingested.

#### Dermal route

While the algorithm remains the same, for articles the default TL in the TRA is reduced to 0.001 except for the following scenarios which retain 0.01: toys (cuddly toy); car seat, chair, flooring; diapers; sanitary towels; tissues, paper towels, wet tissues, toilet paper. The reduced thickness of a uniform layer of 0.001 cm for some article scenarios has been set in the TRA to account for the reduced mobility of substances in the article matrix (based upon expert judgement and stakeholder consensus). For some ACs intended for prolonged/intensive contact and/or articles where it was reasoned that moisture could be present, the default TL for nonsolid media of 0.01 cm was retained; it was also assumed that the contact might more closely resemble a liquid layer.

Several alternate approaches for estimating dermal exposure via article contact are summarized in Table 3 [10, 35–37]. Delmaar et al. [12] has indicated that the TRA dermal algorithm is not sufficiently conservative for articles, as it neglects replenishment of the substance in the TL considered to be in direct contact with skin from the reservoir within the article. By utilizing an approach that considered diffusivity within the article matrix to be the controlling factor for dermal exposure, these authors generated predictions orders of magnitude higher than the TRA in some cases [12]. However, it is also recognized that this method does not take into account mass transfer to the skin nor uptake within the skin [12, 38]. For the purposes of assessing the TRA, only the mass transfer to the skin is relevant as predictions are for external exposures (as per REACH requirements [14]).

**Table 3:** Article Categories Summary Table—Algorithm Approaches, Model and Data Benchmarking.

ALGORITHMS					
Reference	Algorithm Approach				
Delmaar et al. [12]	An alternate simplified diffusion layer model (for slab-like articles) is proposed that makes use of the average distance that a diffusing molecule will travel in the article matrix within a given time. The diffusion coefficient and the travel time need to be pre-determined. It is then assumed that all of the substance diffused to the article surface is transferred to skin (i.e., no material-skin transfer resistance) and can be dermally absorbed.				
CEM model [35]	Utilizes the diffusion approach of Delmaar along with an absorption fraction.				
EU TGD [17]	Estimates dermal exposure to a non-volatile substance migrating from an article as: [weight of substance on skin per event (g/m^2^)] = [concentration of substance in article (g/m^3)^] X [the article thickness (m)] X [fraction migrating per time] X [exposure time] The guidance indicates that fraction migrating X exposure time must be much less than 1.				
USEPA [36]	From guidance for estimating exposures from contact with materials impregnated with pesticides, exposures are calculated as*:* weight per surface area material type X weight fraction of substance in the material X daily material to skin transfer efficiency x exposed skin surface area.				
Clausen et al. [37]	Clausen et al propose another approach based upon the observation that articles will have a surface film which will be in equilibrium with the substance content in the article matrix, based upon work of Weschler and Nazaroff [50]. Considering both diffusional and then mechanical transfer, a model was developed that resulted in an estimate of about 11% of the TRA v3.1 model. It was indicated, however, that while this model is based to a much greater extent on physicochemical properties than other dermal models, it would need further evaluation.				
Spann et al. [10]	Spaan includes a mass balance approached based upon the thickness of the article and either the skin contact area or the article surface area.				

BENCHMARKING					
Reference	Benchmark Type	Scenarios	Results		
Luccatini et al. [42]	Data from an extensive review of global data for SVOC concentrations in indoor air, dust and consumer products.	Inhalation Route. Data provided for SVOC indoor air concentration in general. Here, the data are compared to all AC inhalation predictions	• SVOC concentrations generally vary from several pg/m^3^ to a few µg/m^3^. Note that multiple articles present in an indoor air environment could contribute to an overall indoor air concentration. • TRA default predictions for the lowest vapor pressure band for articles range from 0.04–59 mg/m^3^: - The 0.04 mg/m^3^ value is associated with tissues, toilet paper, paper towels. - The next lowest value is 0.3 mg/m^3^.		
Data included in SI Table 9.1	Data: Chamber measurements of emissions for TV sets and computer monitors	Inhalation Route. AC13 subcategory “plastics larger articles” includes personal computers.	• Assuming the largest individual chemical emission (0.3 mg single chemical per monitor per hour, substance with VP > 10 Pa) for the 8-hour TRA scenario (instantaneous release in a 20 m^3^ room), would give an air concentration of 0.1 mg individual substance/m^3^ per event • TRA inhalation prediction of 29700 mg/m^3^ event average for the highest VP band or 29.7 mg/m^3^ for the lowest vapor pressure band.		
Baker et al. [43]	Data	Inhalation Route. Dry cleaned clothes	Upon bringing dry cleaned clothing into a residence: • Residential air concentrations: up to 0.3 mg/m^3^ for perchloroethylene (VP>100 Pa) • Maximum perchloroethylene source strengths ranged from 16–69 mg/hour TRA AC5 clothing predicted air concentration across VP bands: 1.85–1850 mg/m^3^.		
OECD [40]	Modeled. Summary of results of multiple modeling studies.	Oral Route. AC5:Fabrics, textiles and apparel: Bedding, mattress (OECD flexible foam) AC5 Toys (cuddly toy) (OECD textiles on toys) AC8: Paper articles- printed papers (OECD childrens books) AC13: Plastic articles Toys (OECD plastic toys)	Comparison of oral exposure estimates- mg/kg/day		
					OECD range of typical to
				TRA	worst case across studies
			AC5 Bedding	0.1	<0.001–0.08, *N* = 2
			AC5 Toys	1	<0.001–0.001, *N* = 1
			AC8 Printed paper	3	<0.001, *N* = 1
			AC13 Toys	0.43	<0.001–0.25, *N* = 10
Aurisano et al. [41]	Modeled based upon database of measured chemical migration rates into saliva; used average and 99^th^ percentile mouthing times for specific article, for age groups 3-<6 months and 2-<3 years	Oral Route. Doll (Polyvinyl Chloride-PVC, Polypropylene -PP, Wood), Pacifier (Silicone or Ethylene-Vinyl Acetate -EVA) AC5 Toys (cuddly) AC10 Toys (rubber) AC11 Toys (wood AC13 Toys (plastic including teething rings)	Exposure range across scenarios (average and 99^th^ percentile mouthing times, pacifiers and dolls, both age groups): 4 x 10^–11^ − 2.52 x 10^−1^ mg/kg BW/day; highest exposures in plastic products For comparison, TRA results (mg/kg/day): Toys, cuddly, rubber or wood : 1 Toys, plastic: 0.43		
Delmaar et al. [12]	Modeled: Delmaar diffusivity approach and TRA	Dermal Route. AC5 Fabrics: Textile flooring, Clothing, bedding AC10: Rubber articles, Flooring,Handles, Foot wear AC11 Wood Articles, Flooring, Furniture AC13: Plastic articles, Flooring,Small articles	• Compares the amount emitted (g/event) based upon a diffusion and diffusion layer model with calculated g emitted from TRA (exposure in mg/kg bw/scenario x 60 kg bw = mg emitted) for 10 scenarios. • TRA generally has the lowest exposure prediction by about 2 orders of magnitude, although if recalculated for small plastic articles (0.18 g/event) would be similar to the highest value diffusivity-based value for this scenario.		
Spaan et al. [10]	TRA Mass Balance Approach Diffusivity Approach	Dermal Route: AC5 Fabrics-clothing AC8: Paper articles – printed paper AC13 Plastic articles-flooring	See Figs. SI-9.1–9.3 Dermal Exposure. Presents results for 3 article scenarios utilizing the TRA, as mass balance approach and the diffusivity approach. The TRA run with defaults was most conservative in 2 cases (T-shirt and printed paper) and 2^nd^ most conservative for flooring (mass balance was more conservative).		
Estimate developed using USEPA [36] approach	Modeling	Dermal Route. AC5 Fabrics-flooring	Dermal exposure estimates via carpeting were developed based upon guidance for estimating exposures from contact with materials impregnated with pesticides (USEPA 2012) (details in SI). While this approach has limitations, it is expected to yield conservative dermal exposures for materials evaluated in the EPA document; the default material-to-skin transfer efficiency rates are based on data from carpets and hard surfaces that have had a chemical applied to their external surface rather than incorporated into the article matrix. Dermal Exposure Estimates from Carpet: • USEPA approach: 6300 mg/day or 105 mg/kg/day. • TRA estimate: 8750 mg/day or 146 mg/kg/day. • We note that if the higher daily skin transfer efficiency value of 0.08 was applied to the diffusion layer model estimates of Delmaar, the results would be similar to or an order of magnitude greater than TRA predictions. • We also observed that the application of a daily transfer efficiency has limitations as in reality the value will depend upon the amount present in or on the article, the nature of the article and of the skin contact, and in this case is a relative value expressed as a fraction of the total.		
Estimate developed based upon Abdallah and Harrad [45]	Data	Dermal Route. Clothing and Dust	Abdallah and Harrad experimentally determined the percentage of applied dose that was dermally absorbed for brominated flame retardants in dust and fabric applied to skin (0.5–3.8%), as well as the percent of applied dose that remained within the skin for a 24-hour exposure period (3–12.5% based upon Abdallah Fig. 1), resulting in total absorbed + in skin fractions of 5.3–13.5%; the ratio of percent in skin: percent absorbed ranged from 1–26. For fabrics, they estimated absorbed exposure in the 0.06–120 ng/kg body weight range for a 70 kg adult male sitting on a sofa for 4 hours/day in summer. When summed by class, this resulted in total PBDE absorbed exposures of 8.8 ng/kg bw/day and total HBCDs 111 ng/kg/day. By class the sum of absorbed and remaining in skin for PBDEs was 11–13% and for HBCDs from 1–2% to 5–11%. Even with tenfold increases in the exposure estimates to include amount present in skin also (88–1110 ng/kg/day), they would still be orders of magnitude lower than TRA default estimates for dermal exposure from fabrics (146 mg/kg/day for car seat, chair, flooring).		
Estimate developed based upon Bartsch et al. [46]	Data and modeling	Dermal Route. AC13 plastic small articles (hammer handle)	A model analysis was developed based upon the data in Barsch et al., 2016 using both the TRA approach and the diffusivity model (Fig. SI-11.1). This shows that while the diffusivity model predicts greater dermal exposures than the TRA, both values are much higher than the amount that reaches the skin.		
Analysis developed based upon Qian et al. [44]	Data and Modeling	Total all routes and sources for comparison to dermal route and specific product estimates. AC plastic flooring	- An analysis of the US National Health and Nutrition Examination Survey (NHANES) data that converted urinary biomarker concentrations to daily intake reported a 95^th^ percentile DEHP exposure of 20.4 μg/kg/day based upon the 2007–2008 period [44]. - This exposure is about 5 orders of magnitude lower than the Delmaar prediction for flooring, 4–5 orders below the Spaan mass balance prediction, and 3 orders of magnitude lower than the TRA prediction. - This difference is not explained by possible differences in the actual weight fraction of DEHP in flooring and the model analysis. DEHP concentrations range from 2–35% in all matrices including PVC (ECHA [47], Annex 1). The TRA default weight fraction for flooring was 10%. Therefore, even using the lowest weight fraction of DEHP would reduce the TRA estimate by only a factor of 5. - In addition, the biomonitoring estimate represents exposure from all sources not just flooring. - While these data are not for the EU population, similar conclusions regarding the conservative nature of the dermal exposure estimates as compared to measured total exposure would apply for the EU population based upon comparison of these US data with data from the demonstration project of the Consortium to Perform Human Biomonitoring on a European Scale (DEMOCOPHES) for 17 EU countries [51]: Geometric mean (μg/L) ƩDEHP creatinine- adjusted urinary metabolites		
				EU [51]	US [44]
			Children	47.6	26.5
			Mothers	29.2	19.5
			Thus US NHANES 2009–2010 data were found to be lower but within a factor of two as compared to EU data.		

**Fig. 3:**
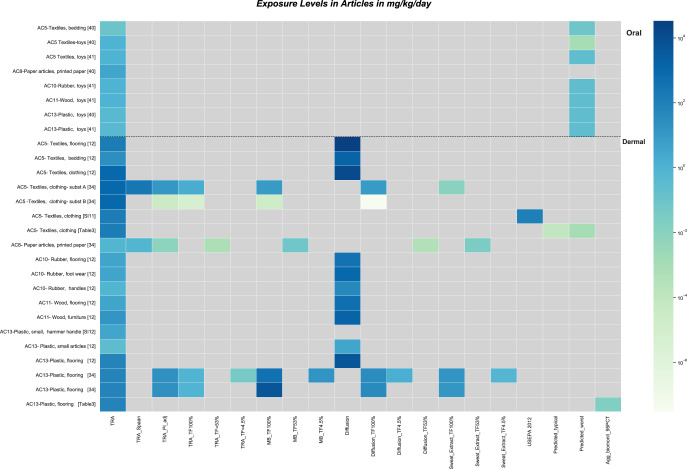
ACs: Comparison of TRA exposure predictions with predictions of other models and available data. Note log scale of coloring. Gray color indicates not assessed (no information). Vertical axis provides article category and source (reference number, table number, or supplemental information section). Horizontal axis provides the name of the model used to generate the prediction, specific model adjustments (PI = product ingredient which is the weight fraction, TF = transfer factor), if the exposure predictions are data-based (if so, whether a typical or upper bound value), and for one substance present in flooring the aggregate exposure estimate of total exposure from all sources based upon NHANES biomonitoring data. Additional details for all are found in Table 3 and/or supporting information.

Information underpins Fig. 3.

Huang et al. [38] reviewed models for near-field exposure pathways of chemicals in consumer products. This assessment included both the TRA and Delmaar approaches for the pathway of transfer of chemicals from within a solid object to skin surface, and authors concluded that this pathway was considered immature as few models were available to predict this transfer or existing models required chemical specific parameters for which adequate prediction methods are not currently available.

Alternate proposed methods are discussed further in the comparison with other modeled results.

#### ACs—total exposure

As for PCs, total exposure also considers dermal, inhalation and oral without conservation of mass balance (i.e., all released to air, yet exposure via dermal contact or ingestion also occur in relevant scenarios).

### ACs—scenario dependent parameterization

Overall data for refining or evaluating parameters used for prediction of article exposures is limited (Table SI-9.1). No SCEDs were identified for any ACs. One study provided some information regarding exposure duration and frequency and use amount for 2 articles [29]. RIVM has a fact sheet for toys which also includes several scenarios included in the TRA, but this fact sheet is from 2002 and most model parameters derive from estimations [39].

#### Oral route

For objects, 10 cm^2^ is a common value used for the surface area of the object placed in the mouth [40], based upon mouth size. The volume of material ingested in the TRA article scenarios ranges from 0.01–0.3 cm^3^, which would be equivalent to ingestion/absorption from a thickness of 0.001–0.03 cm for a 10 cm^2^ area. In comparison with the TRA assumption that 100% of the substance present is ingested, article-to-saliva leaching data for several article types indicates that for nano silver only a fraction of the total content is leached (Table SI-9.1). A recent comprehensive review of measured migration rates of substances from articles into saliva reported a range from 1.7 × 10^–6–33^ μg/10 cm^2^/min [41]. Using these values and a constant 10 cm^2^ contact, to reach the TRA exposure estimates of 0.1–4.3 mg/kg/day across article categories for a 10 kg child, mouthing would need to occur ≫ 24 hours/day based upon the lowest migration rate, and 0.5–22 hours/day for the highest migration rate. The highest migration rate is for a PVC article, whereas the TRA scenario with the lowest predicted exposure is for bedding. The next highest TRA exposure would be reached in about 2 hours with the highest migration rate. As daily mouthing times for individual article categories are typically <1 hour/day [23], these comparisons indicate the TRA should provide conservative values for most substance-material combinations, and the highest substance-material migration rate may yield an estimate similar to the TRA prediction dependent upon the mouthing time associated with the particular scenario. Using predicted migration rates for each substance-material datapoint (*N* = 437), the review authors developed oral exposure predictions for dolls and pacifiers. Highest predicted exposures were 22–253 μg/kg body weight/day for dolls and 6–224 μg/kg/day for pacifiers. The lowest TRA prediction of 100 μg/kg/day for mattress bedding falls within the range of these estimates, and the range of predictions for all other TRA scenarios (430 μg–4300 μg/kg/day) fall above these values.

#### Dermal route

Minimal data were available to evaluate or refine the dermal parameters. For articles, in the TRA the TL is multiplied by the density of the article to assess the amount of substance released per unit area of the article over time. As experimental data for TL is limited for articles, Spaan et al. [10] summarized data on the amount of substance released over time (Table SI-9.1).

Spaan et al. [10] concluded transfer from surface to skin after application of substances to surfaces can be high: up to 100% based upon their measurements of application to glass and aluminum. They estimated transfer from surfaces to skins or gloves for applied substances with unknown binding to be 10–60%. For substances within articles, experiments of wiping show < 10% if expressed as the amount present in the first 10 um. Data were not available to address the effect of longer wiping durations. They conclude that using a factor of 1 (i.e., 100%) with a TL of 10 µm, as is currently used in the TRA, should be a precautionary approach, and that values below 0.1 (i.e., 10%) would likely be more realistic for PVC and printed paper for a 10 µm TL.

Data in Table SI-9.1 for leaching of nano-silver into sweat and for transfer from article surface to wipes supports that only a fraction of total mass present is released.

### ACs—comparison to modeled predictions or measured data

#### Inhalation route

TRA air concentration predictions were compared to SVOC indoor air concentrations in general and two ACs where data were identified. In all cases the TRA predictions exceeded measured concentrations, sometimes by orders of magnitude (Table 3) [42, 43].

#### Oral route

Table 3 and Fig. 3 include comparison of TRA oral exposure estimates for 4 ACs. In all cases the TRA prediction exceeds the worst-case estimate.

#### Dermal route

Modeling and data from several studies are summarized in Table 3 and Fig. 3. The approach of Delmaar provides higher dermal exposure predictions than TRA, generally by two orders of magnitude [12]. Other approaches, however, provide lower predictions with the exception of the Spaan mass balance approach for flooring, based upon consideration of surface area of flooring contacted rather than skin contact area. TRA dermal predictions in all cases exceed absorbed concentrations estimated based upon skin absorption data and total exposure (all sources) based upon biomonitoring data [44] by orders of magnitude (Table 3) [44–46]. While it is recognized that there are a number of studies reporting migration rates into sweat for specific chemical-article combinations, it was beyond the scope of this effort to develop a comprehensive database of migration rates. The TRA scenario-relevant studies which were identified support that the TRA approach provides a conservative exposure prediction.

Overall, limited measured data specific to consumer exposure scenarios are available to help evaluate the TRA article exposure predictions. The comparison of model estimates provides a relative order of performance. For inhalation exposures the TRA has been shown to be conservative (Fig. 1). For oral exposures TRA predictions tend to be most conservative but for a limited analysis (Table 3, Fig. 3) that did not include any human exposure measurements. For dermal exposures, TRA tends to be more conservative than other tools with the exception of the diffusivity approach (Table 3, Fig. 3) and in one case, the mass balance approach based upon transfer of substance from the complete thickness of contacted flooring area. Measured skin absorption or human biomonitoring data which were related to the TRA scenarios suggest that dermal exposure via articles is much less than the TRA model predictions [44–46]. As a pragmatic screening tool, the TRA approach seems reasonable, but additional data would be helpful particularly for understanding exposure potential via article contact.

## Discussion

In general, limited measured data are available to assess the performance of the consumer TRA. Therefore, much of this evaluation was based upon comparison with predictions from other consumer tools or approaches. In the absence of data, however, it becomes a challenge to understand which tool may actually provide more accurate predictions.

When used completely with defaults, TRA always provides a conservative estimate of total exposure as compared to other models or data. This may seem an inappropriate comparison, as for example default weight fractions may be greater than those of a specific analysis, but this finding is actually a very important one. The TRA tool was designed to provide a conservative estimate of exposure to quickly specify conditions of safe use and to target where higher tier refinement may be needed. It is important to note that the tool used with default settings provides conservative estimates for screening chemical risk assessment.

When modified to match the defaults of other models or scenario conditions, the TRA still generally provides a conservative exposure estimate, frequently orders of magnitude more conservative than measured exposures or predicted exposures using other models. However, there were several cases identified where TRA predictions were lower than other tools:

- One was for dermal exposure from tile glue use as estimated using the ConsExpo model (Fig. 2, Tables 2, SI-7.1) [7]. The ConsExpo alternate assumptions of 30 g/min of glue on skin over a 4-hour period (based upon paint rather than adhesive use) led to a higher exposure estimate, but it is not clear whether it is a more appropriate one. Indeed, occupational data for dermal exposure for flooring glue application (2 mg/kg body weight on hands -Table 2) [32] suggests a lower value may be more realistic.
- One was for the tool adjusted to match specific experimental conditions in which fraction released to air was reduced by almost two orders of magnitude along with other adjustments [11]. As designed, for an aerosol use the tool applies a factor of 100% release to air to provide a conservative estimate and yields an exposure prediction which far exceeds the measured conditions. This points out, however, that while the tool is capable of refinement, it is important to evaluate any refinements in context of the exposure scenario as a whole.
- In one case, a dermal exposure route was missing—i.e., for spray paint. Because the inhalation exposure estimate is conservative, the TRA inhalation exposure estimate was of the same magnitude as the ConsExpo total exposure (inhalation + dermal) estimate. Occupational data for spray painting suggests that the TRA inhalation exposure would be greater than the sum of the measured dermal exposure and ConsExpo inhalation exposure. In general, the TRA is meant to provide an estimate of systemic exposure, and so the similarity in total exposure is reassuring. However, for completeness and in the case that a route specific exposure was warranted, adding a dermal component to this scenario may be a consideration. Alternatively, this route could be added using the subcategory option of the TRA.
- For dermal exposure from articles, an alternate diffusivity approach that is more conservative has been suggested [12]. This approach is also implemented, for example, in the USEPA CEM model but also with consideration of an absorption fraction [35]. This is the most significant of the findings and so a focus of the rest of the discussion.

ECHA [47] recommends the diffusivity approach as implemented in USEPA’s CEM model, but also acknowledges that: “The CEM model/method provides a very conservative dermal (oral) exposure estimation, based on infinite diffusion (not limited by the contact medium) and overestimated coefficients, if defaults are used, especially for semi-volatile organic compounds (SVOCs). ECETOC TRA might give more suitable Tier I estimates, but clarification is needed whether this holds across all substances or whether the applicability domain of the TRA is more limited in terms of substance properties”. The diffusion model is also indicated not to be applied to substances of weight fractions >2%, as at higher concentrations the substance may start changing the diffusivity of the matrix [48]. In practice, application of the diffusion model was demonstrated to also require consideration of the partition coefficient to skin, which results in a different ranking of additives according to their relative release potential. External dermal exposure from articles will depend upon the emission from the article itself and the transfer to skin. Internal exposure then depends upon dermal penetration and uptake through the skin.

While the diffusion model provides higher predictions of exposure, it is important to consider if the exposures predicted are grounded in reality. For example, the diffusion layer model for the textile flooring scenario indicates an exposure of >1000 g/event. As diffusion is a concentration-gradient driven process, values this high seem to exceed maximal skin loading capacity and would also result in very short article lifetimes. Daily removal of functional ingredients at this rate would both degrade article characteristics and deplete article mass to a degree not observed in practice. For articles such as flooring, where multiple individuals in a household contact the same article in a given day, the loss of article functionality and mass would be additive. In addition, very high dermal loadings would not necessarily result in proportionally high internal doses; a recent review of skin permeability coefficients indicates that most log Kp values fall between 10^–6^ to 10^–2^ cm/hour for substances with log Kow 10^–4^ to 10^8^ [49].

Plastic flooring, for which diffusivity and mass balance estimates exceeded that of the TRA, was specifically examined. While these approaches result in predictions higher than those of the TRA, there is no data to support that the higher values are more representative of actual exposures. Exposure from sources that are routinely encountered by a large proportion of the population (such as flooring) should be reflected in general population level biomonitoring data. An analysis of the US National Health and Nutrition Examination Survey (NHANES) biomonitoring data for DEHP (the substance used in the Spaan case study) indicates that the estimated oral equivalent intake corresponding to the 95^th^ percentile of biomonitoring total exposure data is 3 orders of magnitude below TRA dermal predictions from flooring and 4–5 orders of magnitude lower than those of Spaan and Delmaar (Table 3). Note, the biomonitoring data represents aggregate exposure from all sources of DEHP.

Data included in this article and found in Clausen et al. [37] suggest that dermal predictions by the TRA method over-predict actual exposures. An alternate approach proposed by Clausen (Table 3) resulted in an estimate of about 11% of the TRA model. It was indicated, however, that while the Clausen proposal is based to a much greater extent on physicochemical properties than other dermal models, it would need further evaluation.

Thus, for dermal exposure from articles, there is no evidence that the diffusivity approach [11] would provide more appropriate exposure estimates than that of the TRA. As Huang has indicated, the assessment of exposure from articles is a less mature field than for non-article consumer products [38]. Limited data are available to evaluate predictive models for consumer exposure via article contact. This need has been recognized and several studies have been sponsored to help advance science in this area, but there remains more to be done. It may be useful to further explore occupational data to better understand factors related to higher dermal loadings, so that this can be taken into account for consumer settings.

It is recognized that scientific developments taken place since the ECETOC TRA development could be useful to improve the accuracy of the tool. In some cases, the level of overestimation begs the question of the appropriate balance between conservatism and utility in a screening level tool. For example, the tool predictions exceed mass balance for scenarios with multiple exposure routes. This evaluation, however, was not to identify areas to improve the accuracy of the tool predictions, but rather to evaluate the likelihood and situations of exposure underprediction when applying it as a screening tool. Indeed, some new aspects have been built into the tool since its first version was released, including an approach to consider frequency and duration of use. Other tools have also been developed, for example EGRET, that provide refined estimates based upon the original TRA tool as well as updated versions of the ConsExpo and USEPA Consumer Exposure Model. It must be recognized that model refinements also typically entail additional data input needs. For a pragmatic screening tool, refinement must be balanced with the importance of having the capability to predict exposures based upon minimal input data, so that a broad range of chemical-scenario combinations can be quickly evaluated. The design of the tool as developed follows the Parsimony Principle, using simple linear algorithms consistent with EU guidance and including the key factors identified in assessing consumer product exposure, e.g. mass used and weight fraction.

Another key finding of this study was the limited measurement data available for benchmarking consumer exposure model predictions. Newer, more streamlined measurement technologies for gathering exposure data such as smart phone apps, sensors and new technologies such as robotics now provide opportunities for future expansion of consumer product exposure data that will be useful for assessing model performance. Systematic assembly and evaluation of data for dermal migration from article contact could provide further insight into approaches for dermal exposure assessment.

## Conclusions

An evaluation of the consumer TRA indicates that predictions exceed measured exposures (when these are available), typically by orders of magnitude, and are generally greater than or similar to those of other exposure tools. For dermal exposure from articles, there is no evidence that an alternate more conservative diffusivity-based approach would provide more appropriate exposure estimates than that of the TRA. For one scenario, that of spray painting, it may be useful to add a dermal exposure route. The ability for users to customize some pre-defined parameters, so that a more realistic exposure scenario results, brings with it user responsibility for justifying refinements. As with any exposure tool, when default values are refined to reflect more specific data, the adjusted values must be appropriate for the situation being modeled and the scenario should be holistically considered as parameters may be correlated. We note that limited data were available to assess the TRA performance, and not all exposure scenarios were covered. Additional measured data would be useful to improve understanding of consumer exposures and relative performance of exposure tools, particularly for ingestion and dermal exposure via article contact.

### Supplementary Information

Reporting Checklist

Supplementary Information

## Data Availability

All the input parameters used in exposure modeling along with the results and measured exposure data used for comparison can be found in the Supplementary Information to this article.

## Footnotes

1 RIVM: the National Institute for Public Health and the Environment (of the Netherlands), BfR: the German Federal Institute for Risk Assessment, Danish EPA: the Danish Environmental Protection Agency, ANSES: the French Agency for Food, Environmental and Occupational Health & Safety, ECHA: the European Chemicals Agency.

2 Since initial development, the possibility to refine exposure estimates based upon the frequency of use as well as the availability of Specific Consumer Exposure Determinants (SCEDS) has been built into the tool. The approach to risk characterization from infrequent exposure applied in the TRA V3.1 tool differs from the approach recommended in ECHA’s IR&CSA Guidance R.15 on Consumer exposure Assessment (ECHA, 2016) and implemented in the current version of the tool in the Chesar platform. A more in-depth review of this approach is currently underway, and a future paper is planned specifically on this aspect. It is not addressed in this paper.
