# Supplementary material for: An assessment of the ECETOC TRA Consumer tool performance as a screening level tool
Source: J Expo Sci Environ Epidemiol. 2023 Jan 21;33(6):980–93. doi: 10.1038/s41370-022-00510-0 (PMC10733139; doi:10.1038/s41370-022-00510-0)
Supplement: Supplementary file 2 — Supplementary Information [file 41370_2022_510_MOESM2_ESM.pdf]

Supplementary Information

Contents: Page

SI1. Inhalation Algorithm 2

SI2. Vapor Pressure Band Analysis for Inhalation Algorithm 5

SI3. Dilution Factor Analysis for Inhalation Algorithm 7

SI4. Dermal Algorithm 9

SI5. Ingestion Algorithm 10

SI6. Product Scenario Default Values 11

SI7. Benchmarking TRA PC Predictions with Modeled or Measured Data 37

SI8. Spray Paint Scenario: Impact of Missing Dermal Route in TRA 49

SI9. Article Scenario Default Values 50

SI10. Benchmarking TRA AC Predictions with Modeled or Measured Data 62

SI11. Benchmarking TRA Dermal Predictions with USEPA 2012 Approach 66

SI12. Comparison of TRA Consumer v.3.1 dermal exposure predictions with an ex vivo dermal migration and permeation data for BaP from Bartsch et al. (2016) 68

SI1. Inhalation Algorithm

In default mode, for all scenarios:

- Frequency of use (events/day) = 1, exception air care instant action aerosol=4
- Inhalation rate = 1.37 m^3/hour (equivalent to 33 m^3/day if sustained)
- Room volume = 20 m^3
- Body weight = 60 kg for adult or 10 kg/child.

Dilution fraction (unitless) = 0.17 – 0.87 (for 8 hours – 0.3 hours) based upon 0.6 ACH for room without active ventilation

Fraction released to air is 1 for sprays and varies by vapor pressure band for non-sprays:

VP Band	Default fraction released to air
>=10 Pa	1
1-10 Pa	0.1
0.1 -1 Pa	0.01
<0.1 Pa	0.001

Inhalation rate, room volume and body weight are all set at constant values. The inhalation rate is more typical of light to moderate activity level (te Biesebeek et al., 2014). If sustained for a 24 hour period, this would be equivalent to 33 m^3/day which exceeds usual daily values of ~20 m^3/day (ECHA 2012 R15 as cited in te Biesebeek et al., 2014). A higher inhalation rate has the impact of making the mg/kg/day exposure prediction more conservative as it increases the predicted intake volume.

Room volume at 20 m^3 is also conservative as it is a fairly small size room, particularly for tasks that are using relatively high volumes of material (te Biesebeek et al., 2014). By using a small room volume, inhalation exposure predictions will be more conservative as the release amount is diluted in a lower volume of air, leading to a higher air concentration.

The body weight is also on the low end for an adult, and for children represents the value for a child of one year. The impact of lower body weight is that exposure expressed on a body weight basis will be increased. An evaluation of the TRA body weight default with data for European populations concluded the TRA values were appropriate defaults for children and adults (Spaan et al., 2014). Indeed, the review suggested the adult default body weight could be made gender specific or raised to 65 kg.

Product ingredient (weight fraction of substance in product formulation), use amount and exposure time vary by scenario. These will therefore be assessed under scenario dependent parameters rather than algorithms.

Saturated Vapor Concentration (SVC):

The maximum air concentration of a substance at room temperature via evaporation is its SVC. If substance-specific molecular weight (MW) and VP are entered, the TRA uses the SVC as an upper bound air concentration. SVC in ppm is calculated using the equation in Hawkins et al.

(1991), and then converted to mg/m^3 by the equation in ACGIH (2009) (detailed in ECETOC 2012). The SVC is applied only to non-aerosol scenarios.

Dilution Factor:

The TRA model does not assume active ventilation but does allow for a typical air exchange rate of 0.6 air changes per hour (ACH) through natural air movement between rooms (even with the doors closed) (RIVM General Fact Sheet). Effectively, this assumption will result in a lower predicted air concentration as the event duration increases (because the total release occurs instantaneously at the start of the event). If units of mg/kg/day are used, the inhalation exposure increases with duration of exposure. The effect of air exchange is greatest for the scenarios of longest duration; as compared to no ventilation, it ranged from about a 6-fold reduction in air concentration for an 8 hour scenario to a reduction by 1.15 for the shortest (20 minute scenario). This relative % decrease would be the same for a substance in any VP band. This default ACH is included in the RIVM ConsExpo model as well (RIVM, 2021).

All but one of the 8-hour scenarios in TRA are for articles; the single product is continuous action air cleaners. Inhalation exposures from these scenarios would be for continuous release over the exposure periods, and the assumption of instantaneous complete release to air at the scenario outset will provide a more conservative estimate than release over the exposure duration in terms of event exposure dose (see Supplementary Information Section 3 for further analysis). Oltmanns et al. (2015) indicated that the ECHA R15 guidance suggested a value of 0.2 ACH based upon the 10th percentile for US data. This recommendation was not found in the most recent ECHA R15 (2016). Using a lower number for ACH would further increase the exposure values as compared to the 0.6 ACH value. Whereas dilution fractions range from 0.17-0.87 based upon the 0.6 ACH (corresponding to 1.15 -6-fold reductions in air concentration), if a value of 0.2 ACH is applied in the TRA dilution fraction calculation resulting values are 0.38-0.95 (corresponding to 1-2.6-fold reductions in air concentration). Thus, air concentrations using the lower ACH would be similar to those of the current default for the shortest scenarios, and about a factor of 2 lower for the longest 8-hour scenarios. The RIVM General Fact Sheet 2014 update retained the 0.6 ACH default. However, the impact of a 0.6ACH as compared to 0.2ACH is further evaluated in Supplementary Information Section 3.

Overall, the assessment of the model algorithm and scenario independent defaults indicates that when run with default values, the TRA model is designed to provide a conservative estimate of inhalation exposure. The assumption of instantaneous release at the start of a scenario and the fraction released based upon vapor pressure banding are conservative assumptions based upon comparison with higher tier model algorithms and comparison with higher tier predictions based upon evaporation rates. To address whether scenario specific predicted exposures are sufficiently conservative, the use amount and weight fraction need to be a conservative representation for the scenario being modeled. These will be addressed under the evaluation of scenario dependent parameters.

References:

ACGIH Threshold Limit Values (TLVs™) for chemical substances and physical agents and Biological Exposure Indices (BEIs™). American Conference of Governmental Industrial Hygienists.

ECETOC. ECETOC TRA version 3: Background and Rationale for the Improvements. Technical Report no. 114, ECETOC- Brussels; 2012.

ECHA. Guidance on Information Requirements and Chemical Safety Assessment, Chapter R.15: Consumer exposure assessment, version 3.0. 2016 European Chemicals Agency, Helsinki Finland.

Hawkins NC, Norwood SK, Rock JC. A strategy for occupational exposure assessment. American Industrial Hygiene Association, Akron, Ohio, USA.

Oltmanns J., Neisel F, Heinemeyer G., Kaiser E., Schneider K. Consumer exposure modelling under REACH: Assessing the defaults. Reg Tox Pharm 2015; 72:222-230.

te Biesebeek JD, MM Nijkamp, BGH Bokkers, SWP Wijnhoven. General Fact Sheet. RIVM Report 090013003/2014. National Institute for Public Health and the Environment (RIVM), Ministry of Health, Welfare and Sport. Bilthoven, the Netherlands.

RIVM National Institute for Public Health and the Environment (of the Netherlands), *Ministry of Health, Welfare and Sport.* ConsExpo Web Consumer Exposure Model. https://www.rivm.nl/en/consexpo. Accessed 7/8/2021.

Spaan S, de Brouwere K, Geerts L, Marquart H. DRESS Guidance document for assessment of dermal exposure of consumers to substances in articles: suggestions for refinement of ECETOC TRA / improvements for the dermal exposure assessment strategy. 2014, Report no. 4.

SI2. Vapor Pressure Band Analysis for Inhalation Algorithm

Four scenarios were run using the ConsExpo (RIVM 2021) model in evaporation mode and compared to the TRA results, using a hypothetical substance of MW = 200 g/mole, logKow = 2, and product ingredient = 0.5 for vapor pressures ranging from 0.0001 to 10000 Pa (Table SI-2.1). The total mass emitted for an event was calculated based upon the mean event concentration and the total air volume during the event based upon the air changes per hour and event time. The mass emitted was then expressed as the fraction of the total mass in the product. The results (Figure SI-2.1, Table SI-2.2) indicate that the TRA vapor pressure bands give predictions 0.5-3.5 orders of magnitude higher than ConsExpo for the range examined. A linear association was observed between release fraction and VP in log scale, up to VP=100Pa. Indeed, the analysis suggests that a release fraction of 0.5 would be conservative for vapor pressure bands up to 100 Pa, whereas the release fraction of 1 is currently applied at 10 Pa and higher. For the lowest vapor pressure band, <0.1 Pa, at the band value TRA predictions are 0.5 orders of magnitude higher than ConsExpo, and as VP continues to decrease the difference between the 2 approaches gets larger (at the lowest VP examined TRA was 3.5 orders of magnitude higher (Figure SI-2.1).

Table SI 2.1. ConsExpo runs with default settings for the scenarios, using a hypothetical substances of MW 200, log Kow=2 and weight fraction = 0.5				
Scenario	Use amount (g)	Room Volume (m^3)	ACH	Exposure time (min)
Painting Scenario, Roller and Brush	1000	20	0.6	132
Cleaning Scenario, All Purpose	400	58	0.5	240
Cleaning Scenario, Floor Stripper	550	58	0.5	90
DIY wood parquet glue – glued to surface	22000	58	0.5	480

Figure SI-2.1

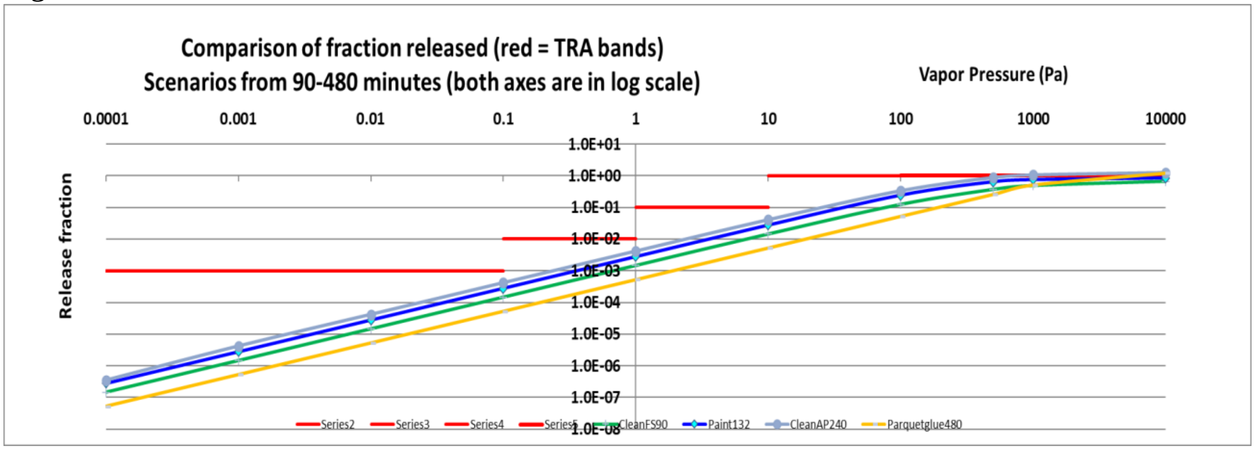

Table SI-2.2	Release Fractions (Fraction of total use amount)				
VP (Pa)		Modeled			
	TRA Band	CleanFS90	Paint132	CleanAP240	Parquetglue480
0.0001	1.0E-03	1.5E-07	2.8E-07	3.5E-07	5.2E-08
0.001	1.0E-03	1.5E-06	2.8E-06	4.2E-06	5.2E-07
0.01	1.0E-03	1.5E-05	2.8E-05	4.2E-05	5.2E-06
0.1	1.0E-02	1.5E-04	2.8E-04	4.2E-04	5.2E-05
**1**	1.0E-01	1.5E-03	2.8E-03	4.1E-03	5.2E-04
10	1.0E+00	1.4E-02	2.7E-02	4.1E-02	5.2E-03
100	1.0E+00	1.2E-01	2.4E-01	3.3E-01	5.2E-02
500	1.0E+00	3.7E-01	6.4E-01	8.7E-01	2.6E-01
1000	1.0E+00	4.9E-01	7.4E-01	1.1E+00	5.1E-01
10000	1.0E+00	6.6E-01	8.4E-01	1.3E+00	1.2E+00

In addition, two studies were identified (ARCADIS 1998, Singer et al., 2006) where sufficient information was available to estimate the release fraction as the amount initially present in the product (summarized in Table SI-2.3). This also supports that the TRA band release fraction is a conservative representation of reality.

Table SI-2.3. Comparison of Emitted Fraction from Monitoring Studies with TRA Release Fraction.			
Substance	VP (Pa)	Fraction Emitted	
		Calculated from Measured Data	TRA
A- Painting	44	0.03	1
B- Painting	1179	0.03	1
C- Cleaning	117	0.11	1

References:

ARCADIS. Testing to determine chemical emissions from paint in support of the EPA Designing Wall Paint for the Indoor Environment Project- Description fo the testing program and results. Draft report prepared by ARCADIS Geraghty & Miller Inc. for the U.S. EPA Office of Pollution Prevention and Toxics under EPA Contract No. 68-W6-0023. 1998

RIVM National Institute for Public Health and the Environment (of the Netherlands), *Ministry of Health, Welfare and Sport.* ConsExpo Web Consumer Exposure Model. https://www.rivm.nl/en/consexpo. Accessed 7/8/2021.

Singer BC, Destaillats H, Hodgson AT, Nazaroff WW. Cleaning products and air fresheners: emissions and resulting concentrations of glycol ethers and terpenoids. Indoor Air 2006; 16(3):179-191.

SI3. Dilution Factor Analysis for Inhalation Algorithm

The ConsExpo (RIVM 2021) model was run for a hypothetical Chemical, MW 200, vapor pressure 200 Pa in evaporation more. The ConsExpo runs were not capped based upon saturated vapor concentration but all TRA calculated values were below SVC, so there should not have been a need to do so.

The impact of dilution by ventilation will increase over exposure duration. Therefore, the analysis was done based upon the longest duration PC scenario in the TRA: "air care, continuous action solid and liquid", with defaults of 0.1 weight fraction, 50 g product, 8 hour duration, 20 m^3 room , 1.37 m^3/hour inhalation rate, and 60 kg body weight. For the ConsExpo evaporation emission estimate a 7 cm^2 surface area was used.

Table SI-3.1 Results of Modeling Runs				
Scenario	ACH	time	Mean event mg/m^3	External event dose mg/kg/day
TRA continuous air care (instantaneous release)	0.6	8 hours	43.1	7.88
ConsExpo Predictions:				
Instantaneous mode	0	8 hours	250	46
Instantaneous mode	0.6	8 hours	52	9.4
Instantaneous mode	0.6	5 hours	79	9
Instantaneous mode	0.6	4 hours	95	8.7
Instantaneous mode	0.6	2 hours	150	6.6
Constant rate mode	0	8 hours	130	23
Constant rate mode	0.2	8 hours	78	14
Constant rate mode	0.2	5 hours	92	10
Constant rate mode	0.2	4 hours	97	8.9
Constant rate mode	0.2	2 hours	110	5
Constant rate mode	0.6	8 hours	41	7.5
Evaporation mode	0	8 hours	2.2	0.4
Evaporation mode	0.2	8 hr release/exposure	1.4	0.3
Evaporation mode	0.6	8 hr release/exposure	0.7	0.1

Blue color denotes 0.6 ACH runs. Yellow highlighting denotes where 0.2 ACH runs assuming a constant emission rate match 0.6 ACH runs assuming instantaneous emission; for longer periods the 0.2 ACH leads to higher exposure predictions.

The impact of ventilation depends upon the exposure metric used and the model chosen for exposure calculations (Table SI-3.1, Figure SI-3.1.)

- ConsExpo in instantaneous release mode gave similar but slightly higher predictions for both mean event air concentration and external event dose than TRA.
- Given same ACH and time, instantaneous mode predictions are higher for both mean event air concentration (mg/m^3) and external exposure dose (mg/kg/day) as compared to continuous or evaporation mode.
- For the instantaneous release model at a given ACH, as the event duration increased the mean event concentration decreased because of dilution by ventilation, but the external event dose increased (as the longer exposure period results in a greater volume of air and airborne substance inhaled even though the mean air concentration is lower). This is seen through the range of TRA scenario durations (up to 8 hours).
- Predictions for 0.6ACH instantaneous mode were higher for both metrics by orders of magnitude as compared to evaporation mode predictions at 0.2 ACH
- Predictions for 0.6 ACH instantaneous mode were also compared to continuous release at 0.2ACH. The ACH instantaneous mode predictions were higher for both metrics for durations up to 4 hours. After 4 hours, the 0.2ACH continuous release mode predictions became higher for both metrics. At the longest duration of 8 hours, instantaneous mode with 0.6 ACH predictions for both mg/m^3 and external event dose mg/kg/day were within a factor of 1.5 of the continuous emission 0.2 ACH predictions.

Figure SI-3.1.

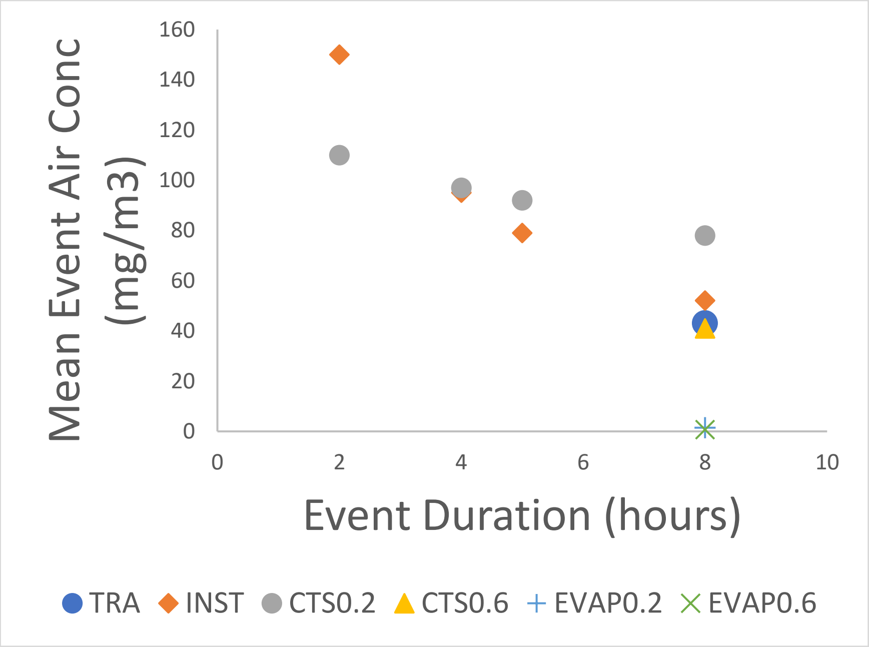

Reference:

RIVM National Institute for Public Health and the Environment (of the Netherlands), *Ministry of Health, Welfare and Sport.* ConsExpo Web Consumer Exposure Model. https://www.rivm.nl/en/consexpo. Accessed 7/8/2021.

SI4. Dermal Algorithm

In default mode, for all scenarios:

- Transfer factor = 1
- Frequency of use = 1
- Density = 1
- Thickness layer = 0.01 for all product scenarios except 0.001 for: air care, continuous action, solid

Product ingredient (weight fraction) and contact area vary by scenario.

With default values, the algorithm will provide a conservative exposure if the parameter defaults are conservative, as it considers that 100% of the substance in contact with skin will be absorbed- that is, the maximal absorption amount. The actual level of conservativeness in the product exposure prediction, however, will depend upon the level of conservatism in the skin contact area, product ingredient and thickness layer (TL) values.

SI5. Ingestion Algorithm

Within the TRA only 3 product categories have an oral route, all are solids or semi-solids: finger paint, putties and clay, fertilizers-lawn and garden preparations. For all scenarios, the:

- Transfer Factor = 1
- Frequency of use =1
- Density = 1

Body weight = 10 kg for all product scenarios (assumption child)

The calculation is conservative as it considers that 100% of the amount ingested is absorbed, but in order to understand if the model prediction is conservative the scenario specific weight fraction and volume ingested need to be assessed. These will be addressed under the parameterization section.

Alternate algorithms have been used for inadvertent ingestion via child mouthing (OECD, 2019). All of these algorithms include the capability for further refinement of the amount ingested by considering migration or mouthing time or other factors, whereas the TRA algorithm assumes that 100% of the amount placed in the mouth is ingested.

Reference:

Office for Economic Co-operation and Development. Estimating mouthing exposure in children – compilation of case studies. Series on Testing and Assessment No. 306. Environment Directorate, Joint meeting of the chemicals committee and the working party on chemicals, pesticides and biotechnology. ENV/JM/MONO(2019)24. 2019

SI6. Product Scenario Default Values

Scenario dependent parameters are summarized in Table SI-6.1, and TRA values are compared with alternate sources of data in Table SI-6.2.

Table SI-6.1. Scenario Dependent Parameters for Consumer TRA (applies to both PC and AC categories).

Parameter	Exposure Route		
	Inhalation	Dermal	Oral
Product Ingredient	X	X	X
Use Frequency	X	X	X
Use Amount	X		
Exposure Time	X		
Room Size (SCED change)	X		
Dilution Fraction (SCED change)	X		
Contact Area		X	
Volume Swallowed			X

Scenario Specific Parameters that apply to D,O,I

Product ingredient weight fraction, when provided, was equal to or lower than the TRA default with the exception of CONCAWE SCED scenarios (PC13 Fuels and all subcategories, and PC 24 Lubricants and Greases subcategory liquid, filling passenger vehicle engine with lubricant). For all CONCAWE SCEDs, the product ingredient weight fraction was increased from 0.5 to 1.0.

Use frequency in the TRA is set at 1/day with the single exception of aircare instant action aerosol spray which is set at 4/day. Previous reviews have shown this to be in general a conservative value as products are generally used once per day or less frequently (CONCAWE 2017, CEPE 2017, ter Berg 2007). One instance, however, was identified where frequency may be more than once per day (values up to 2/day provided) and that was dishwashing soap (AISE 2017, Schneider et al., 2019). For the aircare instant action aerosol spray the TRA value of 4/day is reduced to 2/day in the AISE SCED.

Scenario Specific Parameters that apply to I:

Use amount: Alternate values were the same or lower than those in the TRA with the following exceptions:

- For paints and lacquers TRA uses a default of 1300 g. CEPE (2017), Schneider et al., (2019), and ConsExpo all provide values similar to or lower than this, but Schneider's literature

review indicates that the range of arithmetic means reported for other studies ranged up to 5471 g for latex paints or 44-3052 g other paints. These studies encompassed a range of use conditions and were not specific to 20 m^3 rooms. For products like wall paints, use amount will be related to the coverage area.

- For fillers and putties, TRA uses a default of 1000 g, the CEPE SCED indicates a value of 2000 g, Schneider et al., (2019) reported values from 30 – 4183 g and also indicated ConsExpo had values of 0.25-0.5 g. Within ConsExpo documentation (ter Berg et al., 2007)(not included in Table 6.2), a value of up to 40g is indicated for the filler from tube application scenario.
- For fuels, the TRA default of 5000 g was increased in the CONCAWE SCEDs (CONCAWE 2017) for automotive refueling (liquids, liquified gas, diesel), liquefied gas home space heater and liquids recreational vehicles.
- For laundry and dishwashing products, product use amounts in AISE SCEDs or laundry products and fabric conditions exceeded the TRA default of 50 g. For AISE SCEDs, use amounts were based upon regular not concentrated detergent; weight fractions should be aligned with this.

Inhalation transfer factor. Within the TRA, this factor is autopopulated based upon the VP band that a substance falls in. However, the CONCAWE SCEDs have introduced an inhalation transfer factor that represents the portion of handled substance that becomes available for release into air. For example, when fueling a vehicle only a small portion of the transferred fuel is released to air. Within the TRA tool, this value can be added in place of the VP band specific Inhalation Transfer Factor when creating a new scenario; the new scenario will only utilize the entered value and not be populated by the VP band.

Exposure time: For waterborne latex paint and solvent rich paints exposure time was higher by ~factor of 4 in CEPE SCEDs than the TRA default. CEPE (2017) indicates this time is the 90th percentile from a 2015 painting survey and included exposure time whilst not actually painting. CEPE SCEDs only specify indoor or outdoor locations, they do not specify room size and they do not indicate if there was a correlation between room size (i.e., paint area) and exposure time. Values reported in Schneider et al. (2019) were more consistent with the TRA value. For removers and plasters and floor equalizers, CEPE SCED exposure times were twice that of the TRA. Many of the other scenarios had exposure times much lower than the value used in the TRA.

Room Size and ACH (which impacts dilution fraction) remain unchanged across all scenarios in the TRA. In the CONCAWE SCEDs, however, these values are changed to reflect product use that occurs in a garage or outdoors rather than within the rooms of a residence.

Overall, TRA general defaults were consistent with a lower tier analysis. In several instances the product use amount may be lower than in other sources, but this amount is also tied into other scenario conditions such as room volume. As noted earlier, the key aspect of model evaluation is ideally how predictions compare to measured data or alternatively to other accepted model predictions. In some instances, default refinements were identified for only specific parameters within the TRA, but did not address all aspects of the equation needed to run the TRA model. The SCEDs however, by following the SCEDs template provide sufficient information to rerun a

scenario. Exposure predictions for the TRA are compared to that of the SCEDs in Figures SI 6.1-6.4. Note the inhalation predictions are based upon a VP falling within the highest VP band; the inhalation fraction for nonaerosols would be reduced at lower VP bands but the reduction would be similar for TRA or SCED. These TRA predictions are also chemical agnostic and so an upper bound based upon SVC has not been implemented as no MW was entered.

- In mg/kg/day, the TRA inhalation predictions are higher for all scenarios except some of the coatings subcategories and the putties and plasters and floor equalizers subcategory- here SCEDs values are generally within a factor of 1.5 of the TRA prediction but up to 1.8 for the fillers and putty scenario. This is due to increases in the exposure duration for the CEPE SCEDs for these scenarios, which result in a greater total volume of air inhaled. In addition, for the putties scenario CEPE provides a use amount of 2000 g and indicates this is unchanged from the TRA, but the TRA default for this category is 1000 g. For the 2 coatings scenarios, CEPE provides a range of weight fractions and predicted exposures are lower than the TRA for the lower weight fraction.
- In mg/m^3, the TRA predicted exposure is higher for all of the CEPE scenarios except the fillers and putties where it is 26000 mg/m^3, 1.8 times the TRA prediction. For other categories(nonCEPE) on a mg/m^3 basis, the TRA predicted air concentration is higher with the exception of 2 laundry SCEDs and trigger spray cleaner (Figures SI 6.2). For the laundry SCEDs, greater amounts are used per event and event duration is shorter, resulting in the higher air concentrations. Since the event duration is shorter, exposure on an mg/kg/day basis remains lower (Figure SI 6.1). For the trigger spray cleaner, while use amount and weight fraction are lower the exposure duration is $1/20^{th}$ of that in the TRA, resulting in a higher predicted air concentration. On a mg/kg/day basis the exposure is higher for the TRA for these scenarios. For the scenarios in which SCED-based inhalation exposures were greater either on a mg/kg/day or mg/m^3 basis, total exposures (mg/kg/day sum of inhalation + dermal) were also greater for some of the CEPE scenarios based upon the CEPE upper weight fraction, but TRA predictions were greater as compared to the CEPE lower weight fraction (Figure SI 6.3). For all other categories TRA had the highest total exposure estimates in mg/kg/day.

Scenario Specific Dermal Parameters:

SCEDs values for skin contact area are equal to or less than TRA defaults for all scenarios with the exception of aerosol spray paint. For this scenario the CEPE SCEDs has a value of 428 cm^2, but the TRA does not include a dermal route for this product. None of the SCEDs provided alternative thickness layers.

Dermal Transfer Factor: We note that some SCEDs (FEICA and CONCAWE) provide refined DTFs, reducing the TRA default of 1. As noted within the CONCAWE document, the basis for the DTF needs to be considered when combined with additional parameter refinements. For example, if the refined DTF is based upon a maximum amount on skin for a given scenario, then if other values in the dermal algorithm are refined that could further reduce the amount on the skin, the DTF would need to be adjusted to maintain the appropriate skin contact amount for the scenario.

Exposure predictions for the TRA are compared to those based on SCEDs in Figure SI 6.4 for dermal exposures. TRA dermal predictions were equal to or greater than SCEDs for all scenarios except one. For the aerosol spray coatings subcategory TRA did not include a dermal route, whereas this route was included in the CEPE SCEDs The predicted dermal exposure for this route ranged from 21-36 mg/kg/day, identical to the TRA and CEPE SCEDs dermal predictions for wall paint and solvent paint. This is on the same order of magnitude as the TRA inhalation prediction for the spray paint scenario (47 mg/kg/day).

Scenario Specific Parameters- Oral Route

The volume swallowed is the single scenario dependent variable in the oral equation. No alternate default values were identified for product category scenarios.

Table SI-6.2 Comparative summary of TRA **Product** Scenario Specific Defaults with SCEDs and Other Data. Pink highlighted = TRA. Red text differs from TRA. Yellow highlighted more conservative than TRA. Grey highlighted encompasses TRA.

Relevant Route:			I, D,O,	I	I,D, O	I	I	I	I	I	D	D		
**Descriptor**	**Product Subcategory**	**Reference**	**Product Ingredient fraction (g/g)**	**Amount Product Used per Application (g/event)**	**Frequency of Use (events / day)**	**Fraction Released to Air (g/g)**	**Dilution Fraction (-)**	**Exposure Time (hr)**	**Inhalation Rate (m^3/hr)**	**Room Volume (m^3)**	**Contact Area (cm^2)**	**Transfer Factor (-)**	**Days of User per year (not in TRA)**	**Other comments**
TRA Algorithm			(PI x	A x	FQ x	F x	DF x	ET x	IR x	/ V				
PC1: Adhesives, sealants	Glues, hobby use	TRA	0.3	9	1	1	0.29	4.0	1.37	20	35.7	1		
	Art supply (maybe paint)	Garcia											most non or rare use, users most <2-3/month	
	Glue - children	Garcia											range up to once per day	
	Art supply - children	Garcia											most <= every week	
	Universal glues	FEICA	not pro-vided	9	1	1		4.0			2 finger-tips=15	0.1	55	
	Glues DIY-use (carpet glue, tile glue,	TRA	0.3	15000	1	1	0.22	6.0	1.37	20	857.5	1		

Relevant Route:			I, D,O,	I	I,D, O	I	I	I	I	I	D	D		
**Descriptor**	**Product Subcategory**	**Reference**	**Product Ingredient fraction** (g/g)	**Amount Product Used per Application** (g/event)	**Frequency of Use** (events / day)	**Fraction Released to Air** (g/g)	**Dilution Fraction** (-)	**Exposure Time** (hr)	**Inhalation Rate** (m^3/hr)	**Room Volume** (m^3)	**Contact Area** (cm^2)	**Transfer Factor** (-)	**Days of User per year (not in TRA)**	**Other comments**
	wood parquet glue)													
	Glue DIY	FEICA	not pro-vided	7600	1	1		6.0			palm of hands = 429	0.1	0.5	SCEDs based on parquet floors (higher amount), what is impact of floor covering glue on evapora-tion rate?
	Glue	Garcia											32-40% non or rare use, users most <2-3/month	
	Glue from spray	TRA	0.3	255	1	1	0.29	4.0	1.37	20	35.7	1		
	Spray glue	FEICA	not pro-vided	128	1	1		4.0			Finger-tips= 35.7	0.1	12	
	Sealants	TRA	0.3	390	1	1	0.29	4.0	1.37	20	35.7	1		

Relevant Route:			I, D,O,	I	I,D, O	I	I	I	I	I	D	D		
**Descriptor**	**Product Subcategory**	**Reference**	**Product Ingredient fraction (g/g)**	**Amount Product Used per Application (g/event)**	**Frequency of Use (events / day)**	**Fraction Released to Air (g/g)**	**Dilution Fraction (-)**	**Exposure Time (hr)**	**Inhalation Rate (m^3/hr)**	**Room Volume (m^3)**	**Contact Area (cm^2)**	**Transfer Factor (-)**	**Days of User per year (not in TRA)**	**Other comments**
	Joint sealants	FEICA	not pro-vided	150	1	1					2 finger-tips=15	0.1	3	
PC3:Air care products	Aircare, instant action (aerosol sprays)	TRA	0.5	10	4	1	0.87	0.3	1.37	20				
	Aircare, aerosol	AISE SCED	0.5	10	2	1		0.3						
	Aircare, continuous action (solid & liquid)	TRA	0.1	50	1	1	0.17	8.0	1.37	20	35.7	1		
	Aircare, nonaerosol	AISE SCED	0.1	2.5	1	1		8.0			2 finger-tips=15	1	365	says TRA uses 5 fingertips and they think 2 fingertips
PC9a: Coatings, paints, thinners, removers	Waterborne latex wall paint	TRA	0.5	3750	1	1	0.43	2.2	1.37	20	428.8	1		

Relevant Route:			I, D,O,	I	I,D, O	I	I	I	I	I	D	D		
Descriptor	Product Subcategory	Reference	Product Ingredient fraction (g/g)	Amount Product Used per Application (g/event)	Frequency of Use (events / day)	Fraction Released to Air (g/g)	Dilution Fraction (-)	Exposure Time (hr)	Inhalation Rate (m^3/hr)	Room Volume (m^3)	Contact Area (cm^2)	Transfer Factor (-)	Days of User per year (not in TRA)	Other comments
	Interior wall paint, roller/brush	CEPE	0.3 - 0.5	TRA	TRA =1	TRA =1		8.0			428.8	1	7	
	Exterior wallpaints, roller/brush	CEPE	0.3 - 0.5	TRA	TRA =1	TRA =1		8.0			428.8	1	8	
	Paints and laquers	Schneider		Arithmetic Mean (AM): latex paint 5471, other types 44-3052	AM: latex paint 0.33 per month, other 0.5-4.2 per month			AM: latex paint 5 hours, others 0.4 - 3.2	3.23					
	Wall paint	Garcia											~80% non or rare use, users most <2-5 times per year	
	Solvent rich, high solid,	TRA	0.5	1300	1	1	0.43	2.2	1.37	20	428.8	1		

Relevant Route:			I, D,O,	I	I,D, O	I	I	I	I	I	D	D		
**Descriptor**	**Product Subcategory**	**Reference**	**Product Ingredient fraction (g/g)**	**Amount Product Used per Application (g/event)**	**Frequency of Use (events / day)**	**Fraction Released to Air (g/g)**	**Dilution Fraction (-)**	**Exposure Time (hr)**	**Inhalation Rate (m^3/hr)**	**Room Volume (m^3)**	**Contact Area (cm^2)**	**Transfer Factor (-)**	**Days of User per year (not in TRA)**	**Other comments**
	water borne paint													
	Trim paints interior	CEPE	0.3-0.5	TRA	TRA	TRA		7.0			428.8	1	7	
	Trim paints exterior	CEPE	0.3-0.5	TRA	TRA	TRA		8.0			428.8	1	8	
	Paints and laquers	Schneider		75th percentile 389; (95th 850)	75th pctile= 0.5 per month (95th all =2, user only =4.3)		note 90% of users indicate doors or window or both open	75th percentile 2.3 (95th is 4)					stat. sig correlation between duration and use amount	
	Paints and laquers	Schneider cites Cons-Expo		1000-1300	0.09			2.0						

Relevant Route:			I, D,O,	I	I,D,O	I	I	I	I	I	D	D		
**Descriptor**	**Product Subcategory**	**Reference**	**Product Ingredient fraction** (g/g)	**Amount Product Used per Application** (g/event)	**Frequency of Use** (events / day)	**Fraction Released to Air** (g/g)	**Dilution Fraction** (-)	**Exposure Time** (hr)	**Inhalation Rate** (m^3/hr)	**Room Volume** (m^3)	**Contact Area** (cm^2)	**Transfer Factor** (-)	**Days of User per year (not in TRA)**	**Other comments**
	Paints and laquers	Schneider		AM Schneider study 238, other literature 44 - 5471	AM Schneider study 1.3 times per month, other literature 0.3 - 4.2			AM Schneider study 1.6, other literature 0.4-4						
	Paints and laquers	Schneider		AM latex paint 5471, other types 44-3052	AM latex paint 0.33 per month, other 0.5-4.2 per month			AM latex paint 5 hours, others 0.4 - 3.2	3.23					
	Varnish	Garcia											83-93% rare or non use, users most < 1/year	
	Aerosol spray can	TRA	0.5	300	1	1	0.83	0.3	1.37	20				
	Aerosol spray indoor	CEPE	0.3-0.5	TRA	TRA	TRA		TRA			428.8	tra	5	

Relevant Route:			I, D,O,	I	I,D, O	I	I	I	I	I	D	D		
Descriptor	Product Subcategory	Reference	Product Ingredient fraction (g/g)	Amount Product Used per Application (g/event)	Frequency of Use (events / day)	Fraction Released to Air (g/g)	Dilution Fraction (-)	Exposure Time (hr)	Inhalation Rate (m^3/hr)	Room Volume (m^3)	Contact Area (cm^2)	Transfer Factor (-)	Days of User per year (not in TRA)	Other comments
	Aerosol spray exterior	CEPE	0.3-0.5	TRA	TRA	TRA		TRA			428.8	TRA	8	
	Spray paint	Garcia											86-95% non or rare use, users most <1/year	
	Removers (paint-, glue-, wall paper-, sealant-remover)	TRA	0.9	2000	1	1	0.29	4.0	1.37	20	857.5	1		
	Removers (paint-, glue-, wall paper-, sealant-remover)	CEPE	TRA	TRA	TRA	TRA		8.0			TRA	TRA	7	
`	Removers outdoor	CEPE	TRA	TRA	TRA	TRA		8.0			TRA	TRA	8	
PC9b: Fillers, putties,	Fillers and putty	TRA	1	1000	1	1	0.29	4.0	1.37	20	35.7	1		

Relevant Route:			I, D,O,	I	I,D, O	I	I	I	I	I	D	D		
**Descriptor**	**Product Subcategory**	**Reference**	**Product Ingredient fraction (g/g)**	**Amount Product Used per Application (g/event)**	**Frequency of Use (events / day)**	**Fraction Released to Air (g/g)**	**Dilution Fraction (-)**	**Exposure Time (hr)**	**Inhalation Rate (m^3/hr)**	**Room Volume (m^3)**	**Contact Area (cm^2)**	**Transfer Factor (-)**	**Days of User per year (not in TRA)**	**Other comments**
plasters, modelling clay														
	Fillers and putty indoor	CEPE	0.9	says TRA but 2000	TRA	TRA		TRA			TRA	TRA	7	
	Fillers	Schneider		75th pctile: 30-4183, N-6	75th pctile: 0.5 events/ month (95th pctile all 0.5, users 2)			75th pctile: 0.8 (95th 1.25)						
	Fillers	Schneider cites Cons-Expo		0.25 - 0.5	0.08-0.17 events per month			0.25 - 0.5						
	Plasters and floor equalizers	TRA	1	25000	1	1	0.45	2.0	1.37	20	857.5	1		
	Plasters and floor equalizers	CEPE	0.9	TRA	TRA	TRA		says TRA but =4			TRA	TRA	5	

Relevant Route:			I, D,O,	I	I,D, O	I	I	I	I	I	D	D		
**Descriptor**	**Product Subcategory**	**Reference**	**Product Ingredient fraction** (g/g)	**Amount Product Used per Application** (g/event)	**Frequency of Use** (events / day)	**Fraction Released to Air** (g/g)	**Dilution Fraction** (-)	**Exposure Time** (hr)	**Inhalation Rate** (m^3/hr)	**Room Volume** (m^3)	**Contact Area** (cm^2)	**Transfer Factor** (-)	**Days of User per year (not in TRA)**	**Other comments**
PC9c: Finger paints	Finger paints													
	Finger paints	Garcia											90% adults non or rare use, users most < 2-3/month	
	Finger paints children	Garcia											62-69%% rare or non user, users most <2-3 times per month	
PC12:Fertilizers	Lawn and garden preparations													
	Insecticides	Garcia											53% non or rare use, users most < monthly	
PC13:Fuels	Liquids	TRA	0.5	5000	1	1	0.29	4.0	1.37	20	857.5	1		

Relevant Route:			I, D,O,	I	I,D, O	I	I	I	I	I	D	D		
**Descriptor**	**Product Subcategory**	**Reference**	**Product Ingredient fraction** (g/g)	**Amount Product Used per Application** (g/event)	**Frequency of Use** (events / day)	**Fraction Released to Air** (g/g)	**Dilution Fraction** (-)	**Exposure Time** (hr)	**Inhalation Rate** (m^3/hr)	**Room Volume** (m^3)	**Contact Area** (cm^2)	**Transfer Factor** (-)	**Days of User per year (not in TRA)**	**Other comments**
	liquids, automotive refueling gasoline	CONCAWE	1	37500	TRA	0.002		0.05		100	210.0	0.002	52	note for all CONCAWE inhalation transfer factor is added in the fraction release to air column and is used in place of VP band factor; for outdoor ACH of 2.5 used, 1.5 used for garage (34 m^3)- need to calculate the dilution fraction associated with these
	liquified gas, automotive refueling	CONCAWE	1	43000	TRA	0.0005		0.05		100	0.0		52	
	liquids, automotive refueling, diesel	CONCAWE	1	44000	TRA	0.002		0.05		100	210.0	0.005	52	
	liquids, garden equipment refueling	CONCAWE	1	750	TRA	0.03		0.03		34	210.0	0.001	26	
	liquids, home space heater	CONCAWE	1	3320	TRA	0.02		0.03		TRA =20	210.0	0.001	180	
	liquified gas, home space heater	CONCAWE	1	15000	TRA	0.0005		0.02		TRA =20	0.0		52	
	liquids, recreational vehicles	CONCAWE	1	7500	TRA	0.01		0.02		100	210.0	0.01	52	

Relevant Route:			I, D,O,	I	I,D, O	I	I	I	I	I	D	D		
**Descriptor**	**Product Subcategory**	**Reference**	**Product Ingredient fraction** (g/g)	**Amount Product Used per Application** (g/event)	**Frequency of Use** (events / day)	**Fraction Released to Air** (g/g)	**Dilution Fraction** (-)	**Exposure Time** (hr)	**Inhalation Rate** (m^3/hr)	**Room Volume** (m^3)	**Contact Area** (cm^2)	**Transfer Factor** (-)	**Days of User per year (not in TRA)**	**Other comments**
	liquids, lamp oil	CONCAWE	1	255	TRA	0.05		0.02		20	210.0	0.005		
PC24: Lubricants, greases, and release products	Liquids	TRA	0.5	5000	1	1	0.29	4.0	1.37	20	857.5	1		
	Liquids, filling passenger vehicle engine with lubricant	CONCAWE	1	870	TRA	0.01		0.2		34	480.0	0.001	4	note, CONCAWE uses 34 m^3 garage with 1.5 ACH- dilution fraction needs to be calculated based upon this
PC31: Polishes and wax blends	Polishes, wax / cream (floor, furniture, shoes)	TRA	0.5	550	1	1	0.29	4.0	1.37	20	857.5	1		
	Shoe care	Garcia											20% non or rare users, most <	

Relevant Route:			I, D,O,	I	I,D, O	I	I	I	I	I	D	D		
**Descriptor**	**Product Subcategory**	**Reference**	**Product Ingredient fraction (g/g)**	**Amount Product Used per Application (g/event)**	**Frequency of Use (events / day)**	**Fraction Released to Air (g/g)**	**Dilution Fraction (-)**	**Exposure Time (hr)**	**Inhalation Rate (m^3/hr)**	**Room Volume (m^3)**	**Contact Area (cm^2)**	**Transfer Factor (-)**	**Days of User per year (not in TRA)**	**Other comments**
													every month	
	Polishes and wax blends nonspray	AISE SCED	0.5	550	1	1		4.0			428.0	1	24	
	Polishes, spray (furniture, shoes)	TRA	0.5	135	1	1	0.29	4.0	1.37	20	857.5	1		
	polishes and was blends, spray	AISE SCED	0.5	135	1	1		1.0			428.0	1	8	
	Furniture polish	Garcia											most non or rare users, users most <2-5 times per year	
PC35:Washing and cleaning products	Laundry and dish washing products	TRA	0.6	50	1	1	0.63	1.0	1.37	20	857.5	1		

Relevant Route:			I, D,O,	I	I,D, O	I	I	I	I	I	D	D		
**Descriptor**	**Product Subcategory**	**Reference**	**Product Ingredient fraction (g/g)**	**Amount Product Used per Application (g/event)**	**Frequency of Use (events / day)**	**Fraction Released to Air (g/g)**	**Dilution Fraction (-)**	**Exposure Time (hr)**	**Inhalation Rate (m^3/hr)**	**Room Volume (m^3)**	**Contact Area (cm^2)**	**Transfer Factor (-)**	**Days of User per year (not in TRA)**	**Other comments**
(including solvent based products)														
	Laundry products	AISE SCED	0.3	150	1	1		0.2			857.5	1	235	
	Fabric Conditioners	AISE SCED	0.15	135	1	1		0.02			857.5	1	210	
	Machine dishwashing product	AISE SCED	0.6		1	1					857.5	1	261	Inhalation and oral exposure considered negligible
	Hand dishwashing liquid	AISE SCED	0.3		2			most < 10 min, more < 30 min, some > 1 hour			857.5	1	365	Inhalation and oral exposure considered negligible
	Hand dishwasher liquid	Schneider		75th pctile: 7 (95th 14)	75th pctile : 0.9 (95th 2.8)			75th pctile: 0.3 (95th 0.8)						Statistically sig correlation between duration and use amount

Relevant Route:			I, D,O,	I	I,D, O	I	I	I	I	I	D	D		
**Descriptor**	**Product Subcategory**	**Reference**	**Product Ingredient fraction (g/g)**	**Amount Product Used per Application (g/event)**	**Frequency of Use (events / day)**	**Fraction Released to Air (g/g)**	**Dilution Fraction (-)**	**Exposure Time (hr)**	**Inhalation Rate (m^3/hr)**	**Room Volume (m^3)**	**Contact Area (cm^2)**	**Transfer Factor (-)**	**Days of User per year (not in TRA)**	**Other comments**
	Hand dishwasher liquid	Schneider cites Cons-Expo		7	1.17			0.8						
	Hand dishwasher liquid	Schneider		AM Schneider study: 5.5, other literature 2-7	AM Scneider study: 0.9, other literature 0.6-2.35			AM Schneider study: 0.3, other literature 0.12-0.5						Schneider reports arithmetic mean& standard deviation from studies for the Netherlands, France, EU, South Korea, Switzerland, Lebanon
	Dishwashing detergent	Garcia												% users 92
	Hand washing	Garcia						majority < 10 min, some 20 min or more						frequency majority <= 1/week
	Cleaners, liquids (all purpose cleaners, sanitary products, floor	TRA	0.5	250	1	1	0.83	0.3	1.37	20	857.5	1		

Relevant Route:			I, D,O,	I	I,D, O	I	I	I	I	I	D	D		
**Descriptor**	**Product Subcategory**	**Reference**	**Product Ingredient fraction (g/g)**	**Amount Product Used per Application (g/event)**	**Frequency of Use (events / day)**	**Fraction Released to Air (g/g)**	**Dilution Fraction (-)**	**Exposure Time (hr)**	**Inhalation Rate (m^3/hr)**	**Room Volume (m^3)**	**Contact Area (cm^2)**	**Transfer Factor (-)**	**Days of User per year (not in TRA)**	**Other comments**
	cleaners, glass cleaners, carpet cleaners, metal cleaners)													
	Surface cleaner nonspray	AISE SCED	0.1	110	1	1		0.3			857.5	1	105	
	All purpose cleaner	Garcia						furniture and surfaces most < 30 min, some > 1 hour						% users 73
	Kitchen cleaner liquid	Garcia						kitchen most < 30 min, some > 1 hour						% users 44
	Bathroom liquid cleaner	Garcia												% users 52

		Relevant Route:	I, D,O,	I	I,D, O	I	I	I	I	I	D	D		
**Descriptor**	**Product Subcategory**	**Reference**	**Product Ingredient fraction (g/g)**	**Amount Product Used per Application (g/event)**	**Frequency of Use (events / day)**	**Fraction Released to Air (g/g)**	**Dilution Fraction (-)**	**Exposure Time (hr)**	**Inhalation Rate (m^3/hr)**	**Room Volume (m^3)**	**Contact Area (cm^2)**	**Transfer Factor (-)**	**Days of User per year (not in TRA)**	**Other comments**
	Toilet cleaner	Garcia						toilet bowl most < 10 min, some up to 60 min						% users 85
	Floor cleaner	Garcia												% users 53
	Carpet cleaner	Garcia						most <30 min; some > 1 hour						% users 12
	Glass cleaner	Garcia						windows most 1 hour or more						%users 71
	Wet cleaning wipe	Garcia												% users 24
	Oven cleaner	Garcia												most non or rare users,

Relevant Route:			I, D,O,	I	I,D, O	I	I	I	I	I	D	D		
**Descriptor**	**Product Subcategory**	**Reference**	**Product Ingredient fraction (g/g)**	**Amount Product Used per Application (g/event)**	**Frequency of Use (events / day)**	**Fraction Released to Air (g/g)**	**Dilution Fraction (-)**	**Exposure Time (hr)**	**Inhalation Rate (m^3/hr)**	**Room Volume (m^3)**	**Contact Area (cm^2)**	**Transfer Factor (-)**	**Days of User per year (not in TRA)**	**Other comments**
														users most <2-5 times per year
	Cleaners, trigger sprays (all purpose cleaners, sanitary products, glass cleaners)	TRA	0.2	35	1	1	0.29	4.0	1.37	20	857.5	1		
	Cleaner spray	AISE SCED	0.1	30	1	1		0.2			857.5	1	105	
	Bathroom cleaner spray	Garcia												
	Kitchen cleaner spray	Garcia												% users 44

Figure SI-6.1 TRA and SCEDs Inhalation Predictions (mg/kg/day)

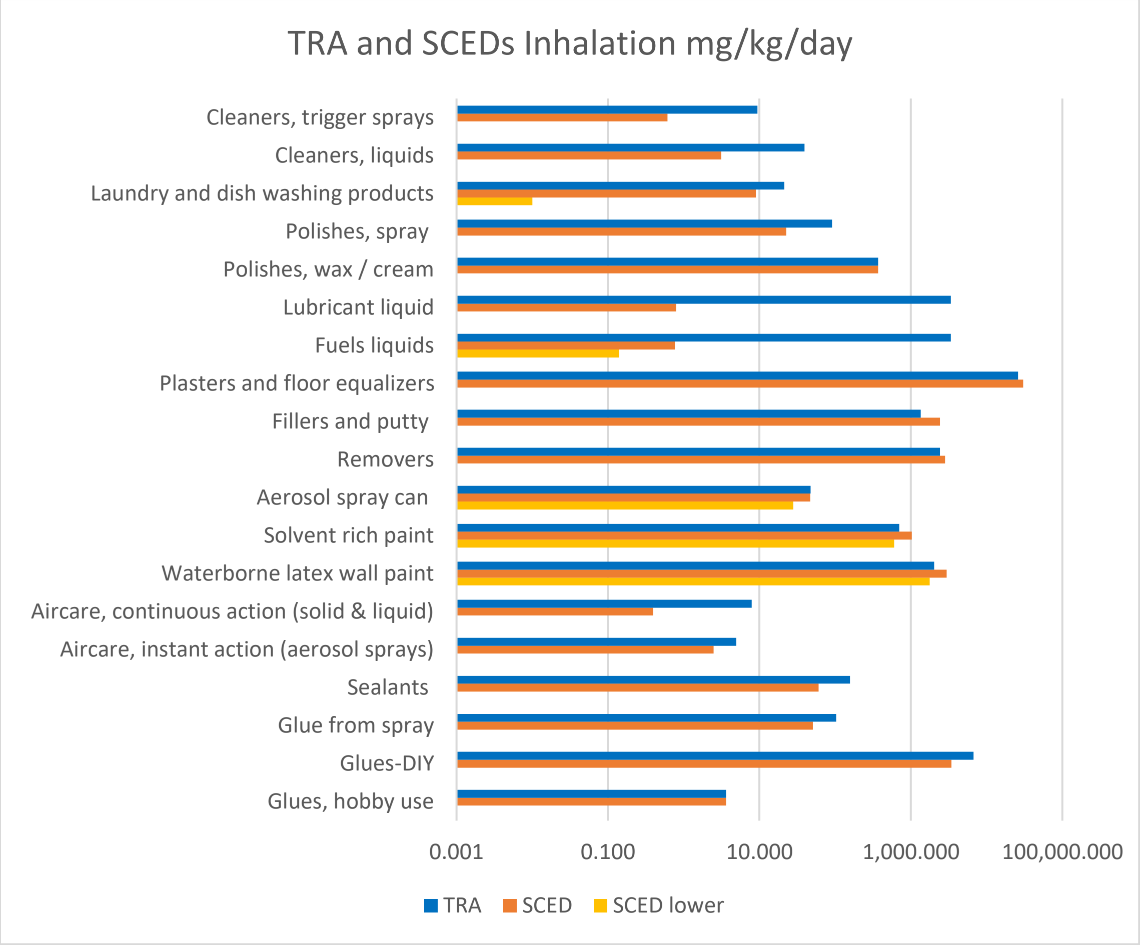

Figure SI-6.2 TRA and SCEDs Inhalation Predictions in mg/m^3

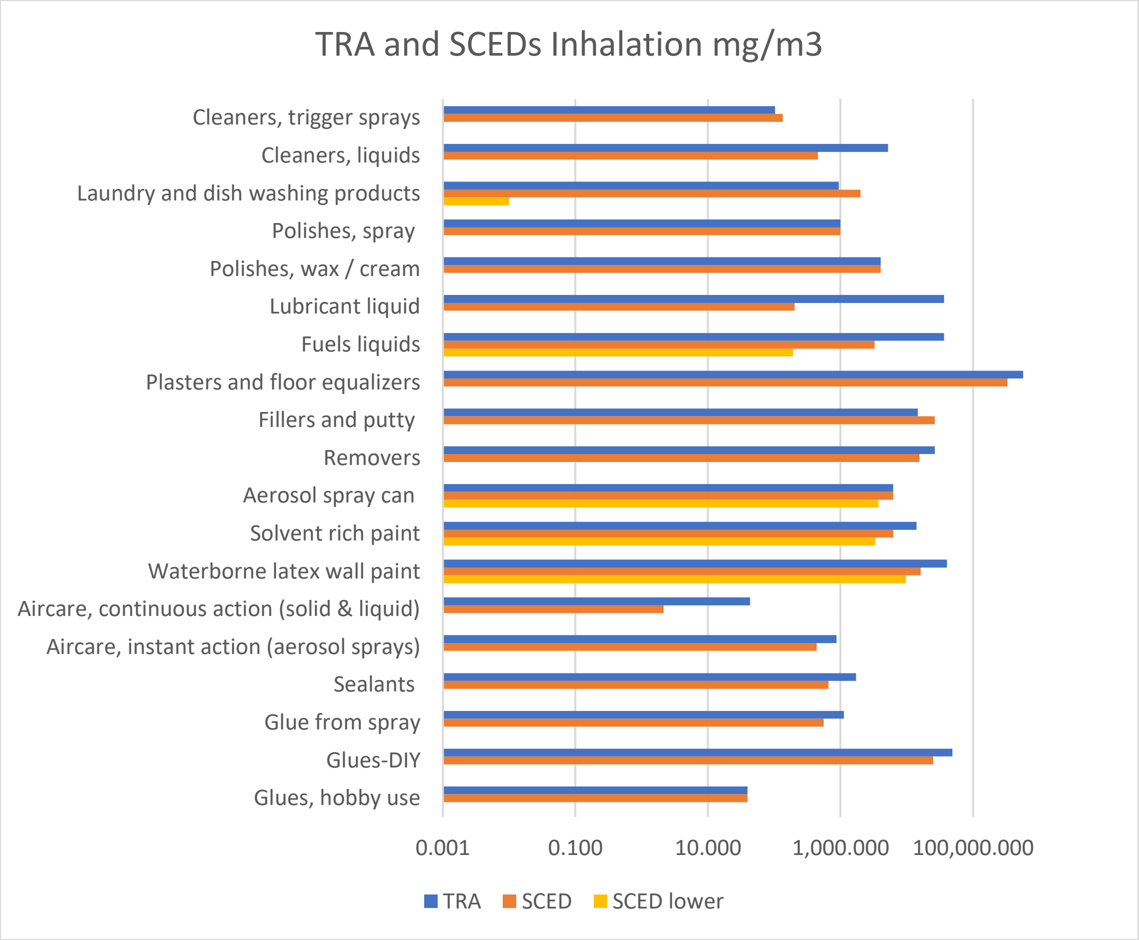

Figure SI-6.3 TRA and SCEDs Total Exposure Per Event

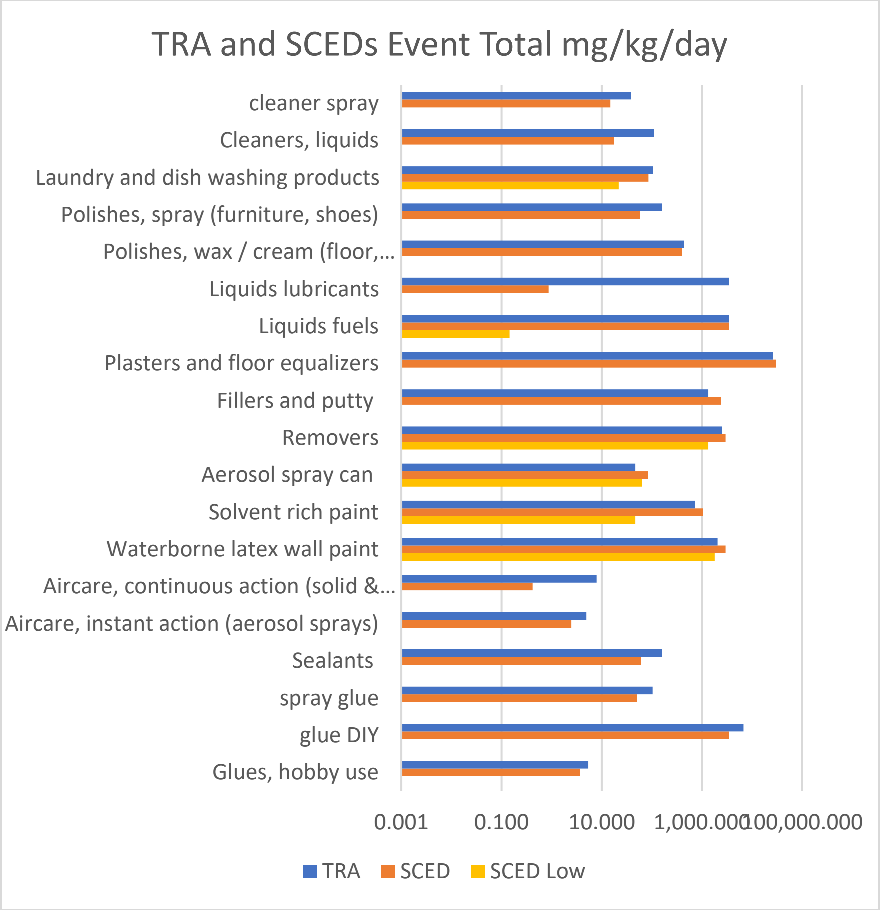

Figure SI-6.4 Dermal Predictions in mg/kg/day

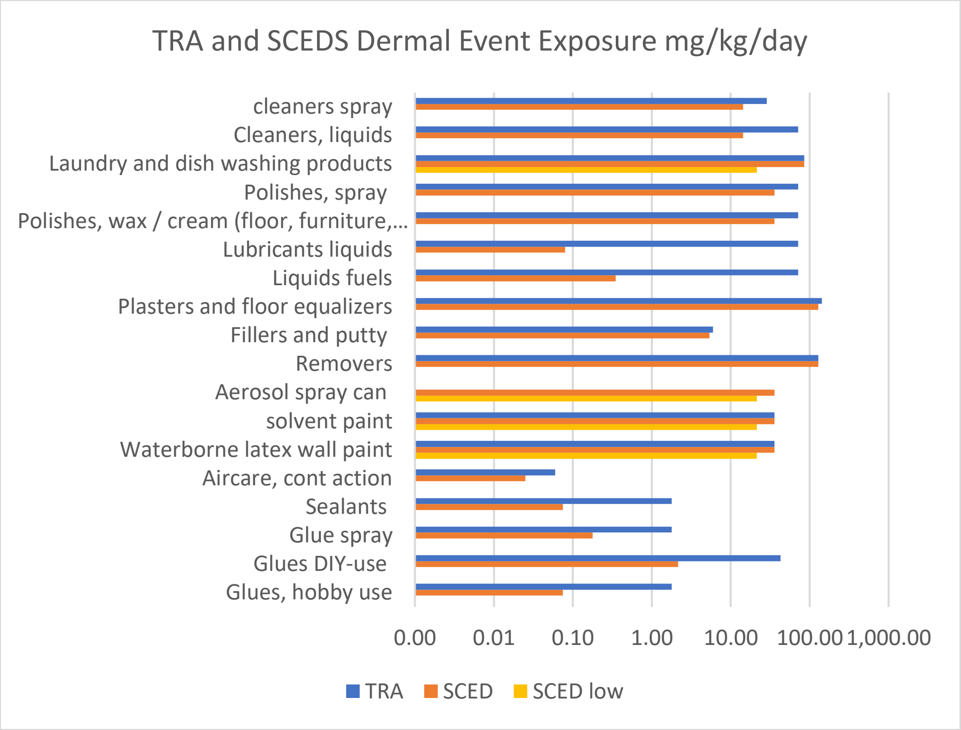

References:

A.I.S.E. / FEA Specific Consumer Exposure Determinants ("SCEDS") Version 1.1 October 2017. Accessed from ECHA Use maps library. https://echa.europa.eu/csr-es-roadmap/use-maps/use-maps-library. Accessed 7/8/2021.

CEPE Specific Consumer Exposure Determinants v1.0 March 2017. Accessed from ECHA Use maps library. https://echa.europa.eu/csr-es-roadmap/use-maps/use-maps-library.

CONCAWE Specific Consumer Exposure Determinants (SCEDs) and Supporting Explanation December 2017. Accessed from ECHA Use maps library. https://echa.europa.eu/csr-es-roadmap/use-maps/use-maps-library.

FEICA Specific Consumer Exposure Determinants (SCEDs) January 2018. Accessed from ECHA Use maps library. https://echa.europa.eu/csr-es-roadmap/use-maps/use-maps-library.

Garcia-Hidalgo E, von Goetz N, Siegrist M, Hungerbühler. Use-patterns of personal care and household cleaning products in Switzerland. Food and Chemical Toxicology 2017; 99: 24-39.

Schneider K, Recke S, Kaiser E, Götte S, Berkefeld H, Lässig J, et al. Consumer behaviour survey for assessing exposure from consumer products: a feasibility study. J Exposure Science and Environmental Epidemiology. 2019; 29(1): 83-94.

Ter Burg W., Bremmer HJ, van Engelen JGM. Do-It-Yourself Products Fact Sheet. 2007. RIVM report 320104007/2007. IRVM, Bilthoven The Netherlands.

SI7. Benchmarking TRA PC Predictions with Modeled or Measured Data

A) Oltmanns et al., 2015:

Oltmanns et al. (2015) compared 4 consumer exposure models (TRA, EGRET, REACT ConsExpo) for six scenarios (bottled glue, tile glue, waterborne wall paint, spray can, all purpose cleaner, glass spray cleaner).

The TRA provided the highest long-term exposure estimate for all scenarios, sometimes by orders of magnitude because it considered each product to be used daily. For short-term exposures (i.e., exposure during the use event), the TRA provided the highest inhalation mg/m^3 exposure estimate for all scenarios except bottled glue, where it was of a similar order of magnitude as the other models, and glass cleaner spraying where it provided a higher estimate than ConsExpo but lower than EGRET. The latter observation is due the longer exposure period in TRA as compared to EGRET, which resulted in a lower time weighted average due to the dilution factor. For bottled glue, ConsExpo had a use amount of 10 g whereas the TRA hobby use scenario default was 9 g; both were deemed reasonable (Oltmanns, 2015). All of these inhalation estimates were substance specific and used a weight fraction adjusted to 0.1. For comparison, TRA default weight fractions are all > 0.1 and result in higher default predictions.

Of the 6 product scenarios examined, TRA had the highest dermal estimates for 5 as modeled by Oltmanns. For tile glue, ConsExpo had a higher dermal exposure as it assumed a dermal contact rate of 30 mg/min over the entire application period of 6 hours, resulting in a 10800 mg total exposure. As noted by Oltmanns and in the ConsExpo documentation the contact rate is based upon data for painting and it is not clear that it is representative for tile glue use. Due to the different nature of these products, it is more likely that glue on the skin, particularly in this amount, would be wiped off as it would be uncomfortable and also make the skin sticky, making working difficult. For the tile glue scenario, the total exposure (inhalation + dermal) of TRA exceeds that of EGRET or ConsExpo (Figure SI-7.1).

For spray paint TRA did not include a dermal route whereas ConsExpo did. The TRA assumed negligible dermal exposure for spray paint because the can would be facing away from the user. ConsExpo predicts a dermal exposure higher than the TRA inhalation exposure for this scenario.

Oltmann's only provided a table of numeric estimates for tile glue, other results need to be estimated from figures. The relative rankings are as indicated above.

Figure SI-7.1. Tile Glue Event Total Exposure- Oltmanns et al., 2015

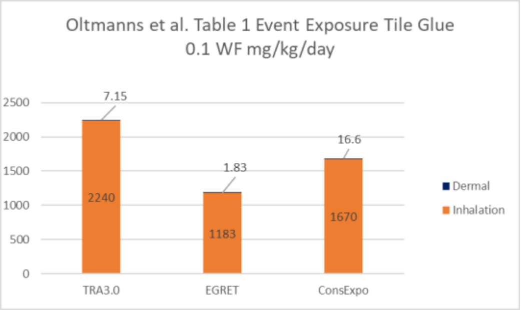

B) ECETOC 2012

ECETOC 2012 provided a comparison of TRA3.0 with ConsExpo4.1, both models run in default mode, (Figures SI-7.1-7.2). This analysis also included comparison to measured air concentration data for 2 substances in solvent coating and 1 substance in a trigger spray cleaner. TRA dermal estimates were greater than all other dermal predictions. TRA predicted air concentrations were orders of magnitude greater than the measured concentrations. The TRA predicted air concentrations were also greater than ConsExpo run with defaults.

Figure SI-7.2 ECETOC 2012 Dermal

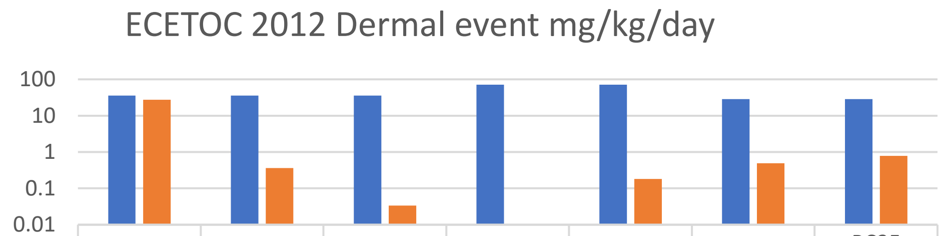

	9a-coatins solvent def	9a-coatins solv substA	9a-coatins solv substB	PC13 auto refuel def	PC35 liquid cleaner def	PC35 trigger spray cleaner def	PC35 trigger spray cleaner SubstC
TRA3.0	35.7	35.7	35.7	71.5	71.5	28.6	28.6
ConsExpo4.1	27.2	0.36	0.034		0.18	0.49	0.78

Figure SI-7.3 ECETOC 2012 Inhalation

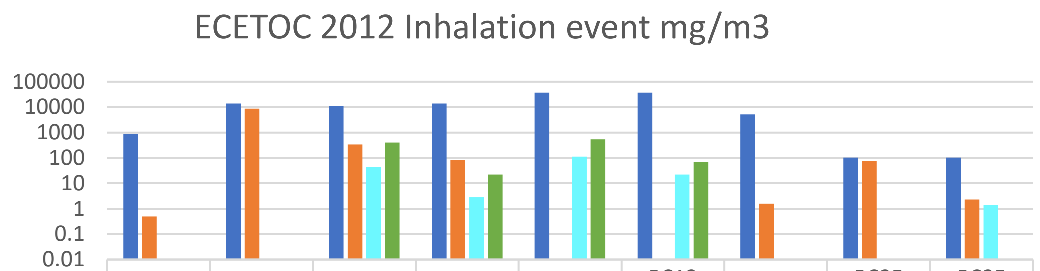

	3 air care inst def	9a-coatins solv def	9a-coatins solv SubstA	9a-coatins solv SubstB	PC13 auto refuel def	PC13 garden equipment use def	PC35 liquid cleaner def	PC35 trigger spray cleaner def	PC35 trigger spray cleaner SubstC
TRA3.0	870	14000	10900	14000	36800	36800	5220	103	103
ConsExpo4.1	0.5	8680	338	80.5			1.6	76.7	2.3
typical data			43.5	2.8	113	22			1.41
data upper			403	22	531	69			

C) Cowan Ellsberry et al. 2020

Cowan Ellsberry et al., 2020 compared TRA3.0 predictions with EGRET, ConsExpo and CEM for 4 scenarios. TRA dermal predictions and inhalation predictions in mg/kg/day exceeded those of the other models (Figures SI-7.4-7.6). The TRA predictions also were greater than those of the 95th percentile of SHEDS-HT, a population level model also included in this analysis.

Figure SI-7.4 Cowan-Ellsberry et al., Dermal

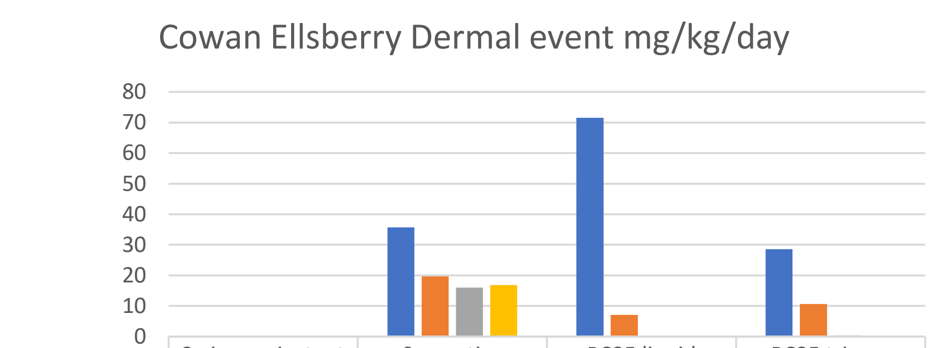

	3 air care instant action	9a-coatins solvent rich	PC35 liquid cleaner	PC35 trigger spray cleaner
TRA3.0		35.7	71.5	28.6
EGRET		19.7	7.15	10.7
ConsExpo		16	0.18	0.37
CEM		16.8	0.24	

Figure SI-7.5 Cowan-Ellsberry et al Inhalation

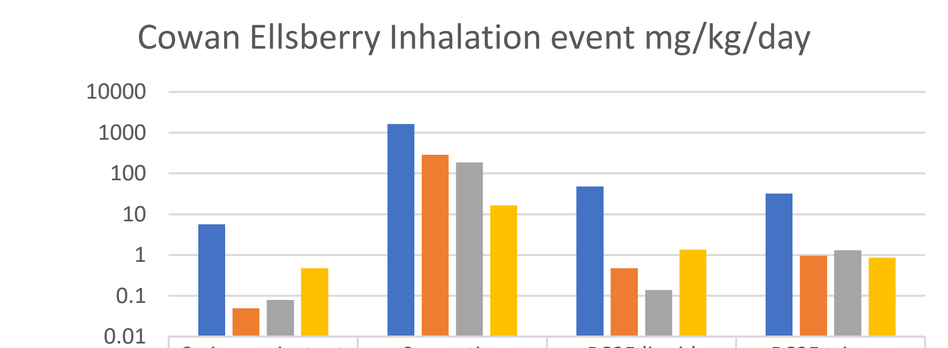

	3 air care instant action	9a-coatins solvent rich	PC35 liquid cleaner	PC35 trigger spray cleaner
TRA3.0	5.7	1630	47.6	32
EGRET	0.05	285.3	0.48	0.97
ConsExpo	0.08	187	0.14	1.3
CEM	0.48	16.6	1.35	0.87

Figure SI-7.6 Cowan Ellsberry et al Total Exposure

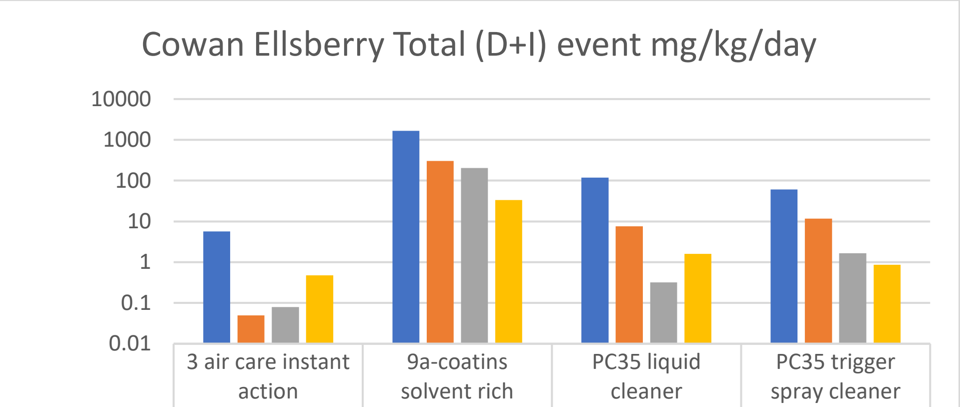

	3 air care instant action	9a-coatins solvent rich	PC35 liquid cleaner	PC35 trigger spray cleaner
TRA3.0	5.7	1665.7	119.1	60.6
EGRET	0.05	305	7.63	11.67
ConsExpo	0.08	203	0.32	1.67
CEM	0.48	33.4	1.59	0.87

D) Feld-Cook et al., 2020

Feld-Cook et al., 2020 collected VOC measurements during a pilot study of a robot painting a wall in a chamber. Figure SI-7.7 summarizes the measured VOC concentrations and modeled VOC concentrations using multiple models. The TRA predictions exceeded the measured data by orders of magnitude and exceeded all other model predictions.

Figure SI-7.7 Feld-Cooks:

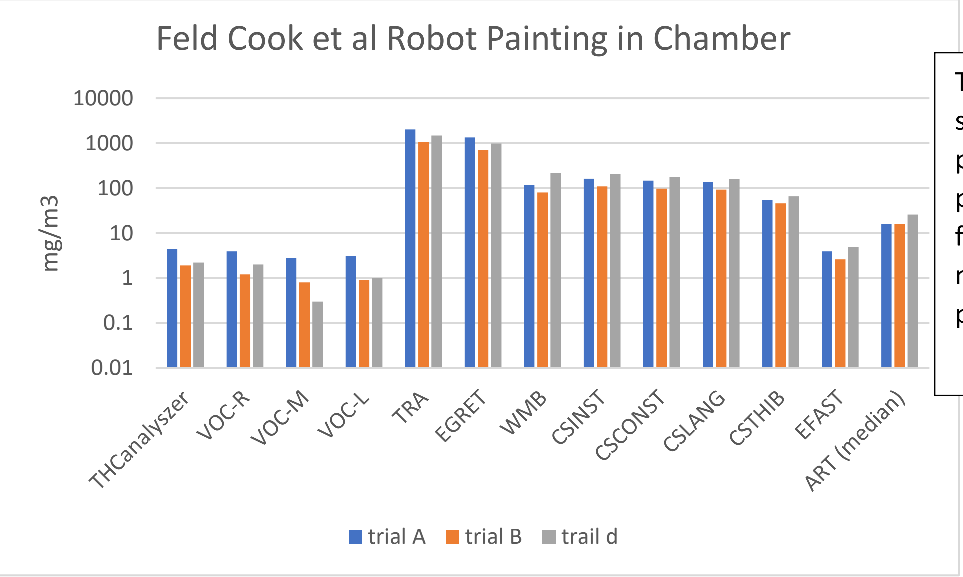

TRA parameterized to scenario; default TRA prediction for wall painting is 40400 mg/m^3 for latex paint and 14000 mg/m^3 for solvent rich paint

E) Steiling et al., 2014

Steiling et al., 2014 provided several modeled estimates for an instant action air freshener spray (Figure SI-7.8). TRA model runs were developed for comparison, using TRA defaults for this scenario and also running the TRA adjusted to match both the 0 and 2 ACH conditions. (Figure 12). The default TRA prediction was orders of magnitude higher than the other predictions; TRA when adjusted to Steiling's conditions yielded similar results, as Steiling's analysis used an equation matching that in the TRA tool.

Figure SI-7.8 Steiling:

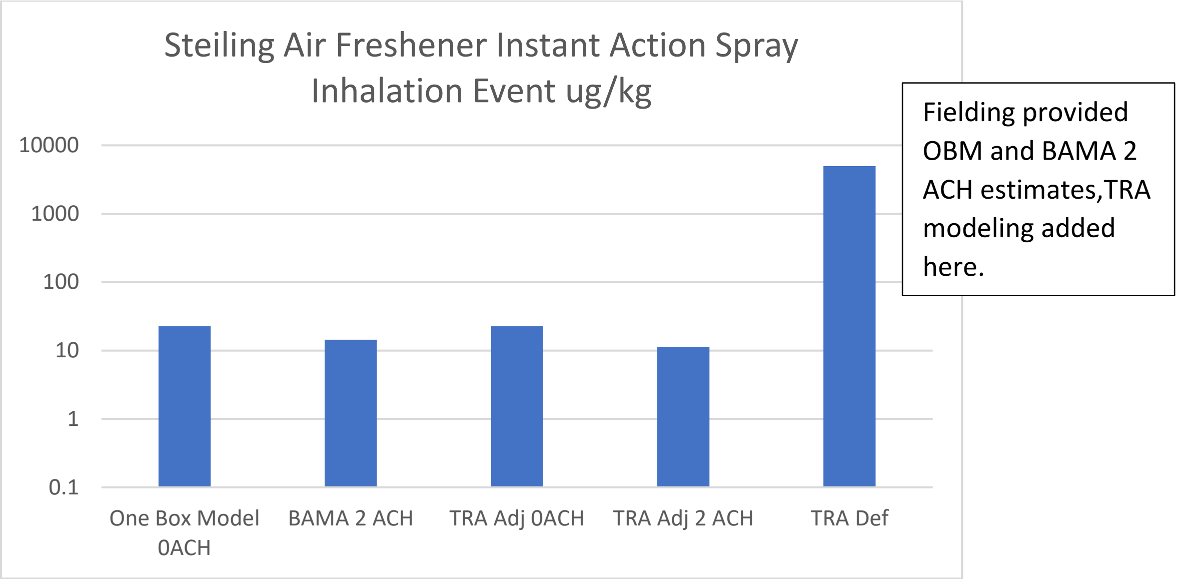

F) Park et al., 2018

Park et al. 2018 measured inhalation concentrations of nano-aerosols during use of consumer sprays and also developed modeled estimates using the TRA, CEM, SprayExpo, ConsExpo Web and ConsExpo nano. The modeled estimates were parameterized to match the experimental conditions. Monitoring data for the mass concentration of aerosols 0.1 – 15 um (fraction of aerosols > 10 um was close to zero) were used to develop an experimental exposure estimate in ng/kg for comparison to the model predictions. Four scenarios were studied varying in amount of product sprayed and air changes per hour (ACH) and modeled: 1-ACH=0. 13.9 g product; 2-ACH=0, 35 g product; 3-ACH=35,13.9 g product 4-ACH=35, 35 g product. Figure SI-7.9 shows the modeled and measure results for total aerosol mass, with the default TRA predictions added in (full TRA defaults and partially adjusted from Park's Supplementary Information). TRA default predictions were orders of magnitude higher than the other model estimates and the estimates based upon aerosol mass monitoring, due to the assumption that 100% of the substance is released to air for aerosol scenarios. Whereas the TRA default for fraction of substance released to air is 1 (representing 100%), the predictions presented in Park were based upon

refinements including reduction of fraction released to 0.01 – 0.06 across scenarios (Park, SI-S2 TRA modeling). Predictions using models adjusted to scenario conditions showed variability in model relative ranking, impacted by both the model approaches as well as which input parameters could be adjusted, and the basis for their adjustment. It is unclear why in some cases, exposure in ng/kg/day decreased for longer durations of the same scenario.

Figure SI-7.9 Park:

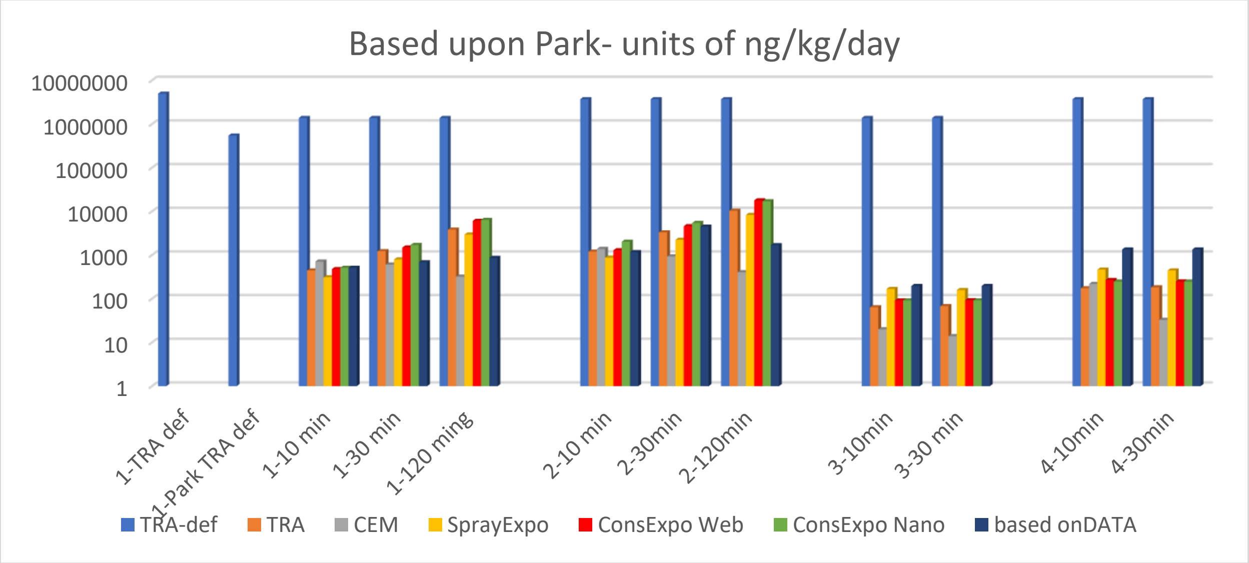

G) Delmaar and Meesters, 2020

Delmaar and Meesters 2020 compared ConsExpo spray predictions to measured data for an all-purpose spray cleaner (Figure SI-7.10). TRA model runs were developed for comparison using only defaults and also adjusted to match the measurement scenario. The TRA predictions exceeded both the measured and ConsExpo modeled air concentrations. Delmar also reran ConsExpo models for the Park (2018) 0 ACH scenarios using a weight fraction range of 0.01 – 0.1, as the 0.1 value was based upon the MSDS concentration and likely represented an upper bound. We compared the peak values from this analysis, which included the peak values from Park to event average values from TRA based upon all defaults and with weight fraction and use amount adjusted to the scenarios. In all cases, the TRA event averages are an order of magnitude greater than the ConsExpo modeled peak or the measured event peak concentration (Figure SI-7.11.

Figure SI-7.10 Delmaar spray:

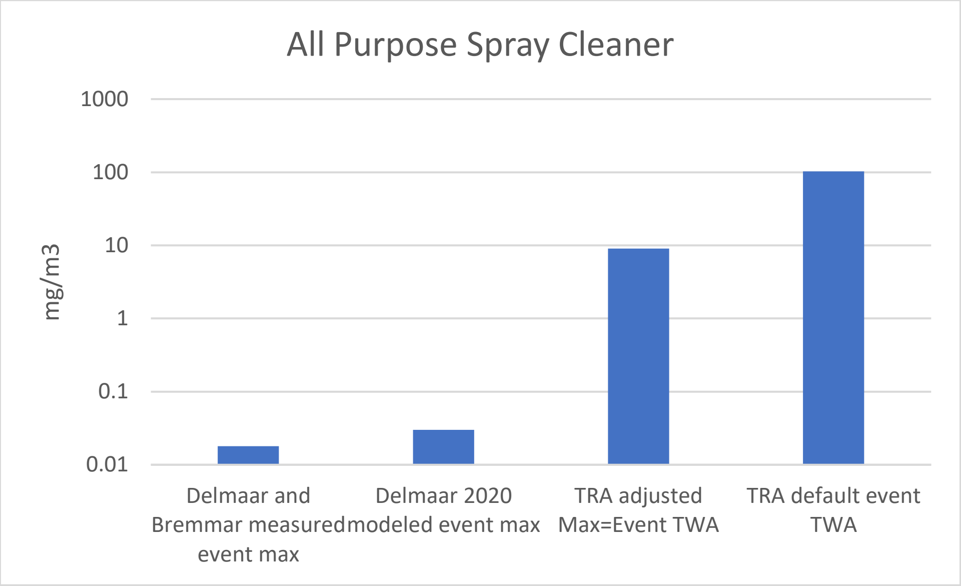

Fig SI-7.11 : Based upon Delmaar Air Freshener Spray, Modeling for Park 0 ACH Scenarios (TRA added in).

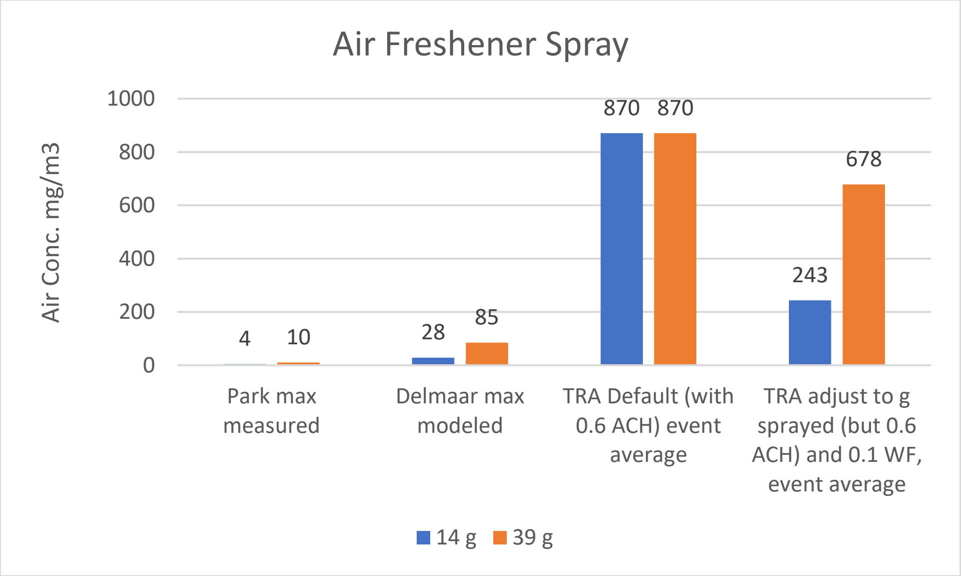

H) EPHECT Emissions Exposure Patterns and Health Effects of Consumer Products in the EU Dimitroulopoulou et al. 2015

The EPHECT study was conducted to improve understanding of household product use patterns, emissions and exposures. Dimitroulopoulou et al. (2015) modeled exposure for several agents in household products based upon the EPHECT data on usage and also measured emissions. The weight fraction present in the products is not provided, but we include here the maximum 30 min air concentration of any of the substances looked at by product category for 0.5 ACH conditions (or the next closest ACH > 0.1), and also the maximum 30 min air concentration resulting from all uses within the household (aggregate exposure). These are summarized in Table SI-7.1 along with TRA default predictions for these categories. The TRA predictions are generally orders of magnitude greater, even as compared to the aggregated exposure air concentration.

Table SI-7.1 Comparison with EPHECT predictions.

	Air concentration estimates (μg/m^3)		
EPHECT Scenario	EPHECT*	TRA	TRA matching scenario
A2 kitchen cleaning agents	1715	5220000	cleaner liquid
A6 furniture polish spray	64	993000	furniture polish spray
A9 air freshener pressurized can1	18	870000	air care, instant action, aerosol spray
A10 passive air freshener	22	43000	air care, continuous action, solid and liquid
A11 electric air freshener liquid 2	38	43000	
All products combined	1716		

*Maximum 30 min concentrations for any of the substances evaluated

I) Several additional studies were identified which provided data relevant for comparison to TRA scenarios are are summarized below.

Table SI-7.2. Additional comparison to measured data

Scenario	Reference	Conditions	Measured Data	TRA Predictions
Floor adhesive	*** Source: Wilke et al., 2004. German BAM research	Low VOC emitting floor glues, measured emissions over 24 hours, exposure	Mean Event Conc.	Mean event conc.

Table SI-7.2. Additional comparison to measured data				
Scenario	Reference	Conditions	Measured Data	TRA Predictions
	report on TVOC and TSVOC emission rates from adhesives and PVC flooring.	estimate developed from this emissions estimate	1.84 mg/m^{3}T VOC+ SVOC	49500 mg/m^3
Air Freshener - gel	Torffs et al		TVOC 1.2 mg/m^3 24 hour peak and 2.2 mg/m^3 1 min peak	TRA Default air freshener solid and liquid 43.1 mg/ m^3
Air freshener - gel	SCHER cites German BUEC Study	Reported TVOC indoor air concentration above background levels in ug/m^3	76-1203 ug/ m^3 [or up to 1.2 mg/ m^3]	
Air freshener-liquid	SCHER cites German BUEC Study	Reported TVOC indoor air concentration above background levels in ug/m^3	78-1956 ug/ m^3 [or up to 2 mg/ m^3]	
Air freshener-electric diffusor	SCHER cites German BUEC Study	Reported TVOC indoor air concentration above background levels in ug/m^3	53-3163 ug/ m^3 [up to 3.2 mg/ m^3]	TRA default air freshener spray 870 mg/ m^3
Air freshener - spray	SCHER cites German BUEC Study	Reported TVOC indoor air concentration above background levels in ug/3m	63-7228 ug/ m^3 [up to 7.3 mg/ m^3]	
Paint remover	Riley et al, also Wallace et al 1989	methylene chloride MW-85, VP = 343 mm Hg X 133.32 Pa/mm Hg = 45728 Pa (USEPA); https://www.epa.gov/sites/production/files/2016-09/documents/methylene-chloride.pdf	Event concentration: 30-1200 ug methylene chloride/ m^3 over 9 hours, one use event per year	TRA VP band default 265000 mg/ m^3 event average; same based upon methylene chloride

Table SI-7.2. Additional comparison to measured data				
Scenario	Reference	Conditions	Measured Data	TRA Predictions
				VP and MW

J) Relevant Occupational Data

Dermal exposure data measured for occupational exposure are provided for scenarios similar to those in the consumer TRA (Table SI-7.3).

Table SI-7.3. Marquart et al. 2006 – Dermal exposure for hands (820 cm^2 for all scenarios), based upon measurements.		
More specific scenarios	Data from studies	Mg per scenario divided by 60 kg to compare with TRA mg/kg prediction
Car body spraying with liquid paint	100 mg/scenario (3 of 30 results> 100 mg)	1.7
Spreading parquet lacquer with a comb	130 mg/scenario (3 of 30 results > 130 mg)	2
Painting window frames with a brush	400 mg/scenario (3 results of 24 > 400 mg)	6.7

References:

Cowan-Ellsberry C, Zaleski RT, Qian H, Greggs W, Jensen E. Perspectives on advancing consumer product exposure models. Journal of Exposure Science and Environmental Epidemiology 2020; 30:856-865.

Delmaar C, Meesters J. 2020. Modeling consumer exposure to spray products: an evaluation of the ConsExpo Web and ConsExpo nano models with experimental data. J Exp Sci Env Epi 2020; 30:878-887.

Dimitroulopoulou C, Trantallidi M, Carrer P, Erthimiou GC, Bartzis JG. EPHECT II: Exposure assessment to household consumer products. Sci Total Env 2015; 536:890-902.

ECETOC. ECETOC TRA version 3: Background and Rationale for the Improvements. Technical Report no. 114, ECETOC- Brussels; 2012.

Feld-Cook E, Shome R, Zaleski RT, Mohan K, Kourtev H, Bekris KE, et al. Exploring the utility of robots in exposure studies. J Exp Sci Env Epi 2021; 31(4): 784-794.

Marquart H., Warren ND, Laitinen J, van Hemmen JJ. Default values for assessment of potential dermal exposure of the hands to industrial chemicals in the scope of regulatory risk assessments. Ann Occup Hyg 2006; 50(5):469-489.

Oltmanns J., Neisel F, Heinemeyer G., Kaiser E., Schneider K. Consumer exposure modelling under REACH: Assessing the defaults. Reg Tox Pharm 2015; 72:222-230.

Park J, Yoon C, Lee K. Comparison of modeled estimates of inhalation exposures to aerosols during use of consumer spray products. Int J Hygiene and Environmental Health 2018; 221:941-950.

Riley DM, Small MJ, Fischhoff B. Modeling methylene chloride exposure-reduction options for home paint-stripper users. J Exposure Analysis and Environmental Epidemiology 2000; 10:240-250

Scientific Committee on Health and Environmental Risks (SCHER). Opinion on the report "Emission of chemicals by air fresheners Tests on 74 consumer products sold in Europe" (BEUC report January 2005). 2006. European Commission Directorate C- Public Health and Risk Assessment.

Steiling W, Bascompta M, Carthew P, Catalano G, Corea N, D'Haese A, et al. Principle considerations for the risk assessment of sprayed consumer products. Toxicology Letters 2014; 227:41-49. https://www.sciencedirect.com/science/article/pii/S037842741400126X

Torfs R., De Brouwere K., Spruyt M., Goelen E., Nickmilder M., Bernard A. *Exposure and Risk Assessment of Air Fresheners.* Health, Food Chain Safety and Environment Section; Brussels, Belgium: 2008. Flemish Institute for Technological Research (VITO) Final Report 2008/IMS/R/222; Produced under authority of the Belgian Federal Public Service

USEPA. Methylene chloride hazard summary. 2000. ; https://www.epa.gov/sites/production/files/2016-09/documents/methylene-chloride.pdf. Accessed 12/1/2021

Wallace LA, Plilizzari E, Hartwell TD, Davis C, Michail L and Whitmore RW. The influence of personal activities on exposure to volatile organic compounds Environ Research 1989; 50(1): 37-55

Wilke O, Jann O, Brödner D. VOC- and SVOC-emission from adhesives, floor coverings and complete floor structures. Indoor Air 14(8): 98-107.

SI8 Spray Paint Scenario: Impact of Missing Dermal Route in TRA

Looking in more detail at the spray paint scenario, the TRA inhalation estimates are more conservative than those of ConsExpo. Exposure values estimated from the figure in Oltmanns et al (2015) for exposure via spray painting with a product of 0.1 weight fraction, are ~1500 mg/m^3 for TRA vs 200 mg/m^3 for ConsExpo. [Note, TRA run with the default 0.5 weight fraction gives an estimate of 6260 mg/m^3.]

Using a default inhalation rate of 1.37 m^3/hour, event duration of 0.3 hours and body weight of 60 kg, total inhalation exposure estimates in mg/kg/event are calculated to be: TRA all defaults- 51.4 mg/kg/day, TRA 0.1 weight fraction - 10.3 mg/kg/day, ConsExpo 0.1 weight fraction 1.4 mg/kg/day. The ConsExpo dermal exposure estimate is 30 mg/kg/day for a 0.1 weight fraction.

The default inhalation TRA value exceeds the sum of ConsExpo inhalation and dermal (31.4 mg/kg/day) but the TRA weight fraction adjusted inhalation value is lower by a factor of ~3.

Measured dermal exposure data for spray painting from the SYSDEA study (Franken et al. 2019) when adjusted to a 0.1 weight fraction, indicate geometric mean total body exposures of 0.4 – 1.2 mg/kg body weight. For a spray painting scenario, clothes are likely to cover much of the body; the SYSDEA hand exposure range was 2.5- 50 mg per event (0.04 -0.8 mg/kg/event for a 60 kg body weight). Adding the upper end of this range to the ConsExpo inhalation estimate provides a total exposure (2.2 mg/kg) lower than the TRA inhalation prediction for a 0.1 weight fraction.

References:

Franken R, Spaan S, Tsakirakis A, Chartzala I, Nikolopoulou D, Anastasiadou P, et al. SysDEA: Systematic analysis of dermal exposure to hazardous chemical agents at the workplace. Final report of project F 2349. BAUA Federal Institute for Occupational Health and Safety, Dortmund, Germany. 2019

Oltmanns J., Neisel F, Heinemeyer G., Kaiser E., Schneider K. Consumer exposure modelling under REACH: Assessing the defaults. Reg Tox Pharm 2015; 72:222-230.

SI9 Article Scenario Default Values

Spaan et al. (2014) reviewed the TRA defaults for articles for both scenario independent and scenario dependent parameters. They indicated that the total body surface area and default values for surface area of body parts based upon them within the TRA tool are appropriate.

Minimal data were available to evaluate or refine the dermal parameters. For articles, the TRA approach the factor Thickness Layer is multiplied by the Density of the article to assess the amount of substance released per unit area of the article over time. Data for amount of substance released per unit area was reviewed but found insufficient to derive suitable defaults for specific exposure events (Spaan et al cites WP 3.2). As experimental data for thickness layer is limited for articles, Spaan et al (2014) took the approach of looking for data to support the amount of substance released over time. Density and transfer factor data for several articles types is included in Table SI 7.1. In addition to the experimental results derived from the DRESS study, Spaan summarized the literature. The Institute of Occupational Medicine transfer efficient database (Gorman Ng et al., 2012) provides a 75th percentile of 14% for transfer of solids from surface to hands and of 58% for transfer from surfaces to gloves; studies were generally for substances applied to surfaces.

The transfer factor, set at 1 for all scenarios in TRA, was evaluated by experimentation. The worst case transfer factor was for substances spiked on smooth glass or aluminum plates, which ranged up to 1, although for some substances the upper value was 0.5. Much lower transfer factors were observed for wipe experiments with real articles, but in these experiments contact with the wipe lasted only a few seconds. When expressed as a fraction of the article mass underneath the wiped surface, transfer was 0.000033-0.006%. When expressed as the mass in a thickness layer of 10 um for the same surface area, the values were 0.05-1.52% relative transfer.

Spaan et al. (2014) concluded transfer from surface to skin after application of substances to surfaces can be high – up to 100% based upon their measurements of application to glass and aluminum. From the literature they estimate transfer from surfaces to skins or gloves for applied substances with unknown binding to be 10-60%. For substance within articles, experiments of wiping show < 10% if expressed as the amount present in the first 10 um; experiments however were for wiping of a few seconds. The effect of longer wiping duration was not addressed. They conclude that using a factor of 1 (i.e., 100%) with a thickness layer of 10 um, as is currently used in the TRA should be a precautionary approach, and that values below 0.1 (i.e., 10%) would likely be more realistic for PVC and printed paper for a 10 uM thickness layer.

The work of Spaan also identified several factors that are not part of the TRA equation but may be used in combination as a refinement of the TRA approach. These include duration of contact, number of contacts per exposure event, number of exposure events, and surface area of article in contact with the skin. The latter suggestion was included in a flooring case study (see SI9 and also article text of this document). The possibility to consider constant contact during a prolonged time or many short duration contacts was also discussed. Both concepts are related to exposures that may be more intense with longer contact and/or multiple contacts which may reload skin. Their relevance would vary by AC type and associated contact behavior. The overall

level of conservativeness currently in the equation as a whole, however, needs to be assessed to understand if modification would be warranted.

Table SI-9.1. Comparative summary of TRA **Article** Scenario Specific Defaults with SCEDs and Other Data. Pink highlighted = TRA. Red text differs from TRA. Yellow highlighted more conservative than TRA. Grey highlighted encompasses TRA.

	**Relevant Route:**		**I, D,O ,**	**I**	**I,D, O**	**I**	**I**	**I**	**I**	**I**	**D**	**D**		
**Descriptor**	**Product Subcategory**	**Reference**	**Product Ingredient fraction**	**Amount Product Used per**	**Frequency of Use**	**Fraction Released to Air**	**Dilution Fraction**	**Exposure Time**	**Inhalation Rate**	**Room Volume**	**Contact Area**	**Transfer Factor**	**Density**	**Other comments**
			**(g/g)**	**(g/event)**	**(events / day)**	**(g/g)**	**(-)**	**(hr)**	**(m^3/hr)**	**(m^3)**	**(cm^2)**	**(-)**	**(g/cm^3)**	
AC codes:		No SCEDS for any article scenario. Limited relevant data from other sources												
AC5: Fabrics, textiles and apparel	Clothing (all kind of materials), towel	TRA										1		
		Spaan											cotton weave: 0.07 - 0.39 g/cm^3, propose 0.2 g/cm^2 as typical value	
		Spaan											other fabrics 0.09 - 0.30 g/cm^3; synthetic aramid used for protective clothing 0.41-0.94	

	Relevant Route:		I, D,O ,	I	I,D, O	I	I	I	I	I	D	D		
**Descriptor**	**Product Subcategory**	**Reference**	**Product Ingredient fraction**	**Amount Product Used per**	**Frequency of Use**	**Fraction Released to Air**	**Dilution Fraction**	**Exposure Time**	**Inhalation Rate**	**Room Volume**	**Contact Area**	**Transfer Factor**	**Density**	**Other comments**
			**(g/g)**	**(g/event)**	**(events / day)**	**(g/g)**	**(-)**	**(hr)**	**(m^3/hr)**	**(m^3)**	**(cm^2)**	**(-)**	**(g/cm^3)**	
													g/cm^3; woven polyester 0.116-0.147 g/cm^3	
		RIVM toy fact sheet										leaching factor azo dyes from textiles, based upon unwashed textiles, ranged from 0.005 - 0.037 g dye per g product		
		RIVM toy fact sheet										leaching 0.011-0.023 ug dye/cm2 after 1 wash, after 29 washes <0.003 - 0.009.		

	Relevant Route:		I, D,O ,	I	I,D, O	I	I	I	I	I	D	D		
Descriptor	Product Subcategory	Reference	Product Ingredient fraction	Amount Product Used per	Frequency of Use	Fraction Released to Air	Dilution Fraction	Exposure Time	Inhalation Rate	Room Volume	Contact Area	Transfer Factor	Density	Other comments
			(g/g)	(g/event)	(events / day)	(g/g)	(-)	(hr)	(m^3/hr)	(m^3)	(cm^2)	(-)	(g/cm^3)	
		RIVM toy fact sheet										leaching parameters of extraction of dye stuffs from textiles range from ND, then 5e-5 to 2e-2 g/g		
		RIVM toy fact sheet										dislodgeable formulation from tent canvas after brushing 0.014- 0.04 ug/cm^2		
	Bedding, mattress	TRA										1		
		Quadros										baby blanket, 110 mg Ag/kg product: amt leached to saliva was 1.2mg/kg or 1.1%; for sweat 4.8 mg/kg or 4.4%; silver transferred		

	**Relevant Route:**		**I, D,O ,**	**I**	**I,D, O**	**I**	**I**	**I**	**I**	**I**	**D**	**D**		
**Descriptor**	**Product Subcategory**	**Reference**	**Product Ingredient fraction**	**Amount Product Used per**	**Frequency of Use**	**Fraction Released to Air**	**Dilution Fraction**	**Exposure Time**	**Inhalation Rate**	**Room Volume**	**Contact Area**	**Transfer Factor**	**Density**	**Other comments**
			**(g/g)**	**(g/event)**	**(events / day)**	**(g/g)**	**(-)**	**(hr)**	**(m^3/hr)**	**(m^3)**	**(cm^2)**	**(-)**	**(g/cm^3)**	
												onto dermal wipe was 23 ug/m^2		
	Toys (cuddly toy)											1		
		Quadros										plush toy interior foam, 48 mg Ag/kg product: amt leached to saliva was 1.77mg/kg or 3.7%; for sweat 18.5 mg/kg or 38%		
		Quadros										plush toy exterior foam, 0.6 mg Ag/kg product: amt leached to saliva was 0.03 mg/kg or 5.6%; for sweat 0.14 mg/kg or 2.6%; Ag		

	**Relevant Route:**		**I, D,O ,**	**I**	**I,D, O**	**I**	**I**	**I**	**I**	**I**	**D**	**D**		
**Descriptor**	**Product Subcategory**	**Reference**	**Product Ingredient fraction**	**Amount Product Used per**	**Frequency of Use**	**Fraction Released to Air**	**Dilution Fraction**	**Exposure Time**	**Inhalation Rate**	**Room Volume**	**Contact Area**	**Transfer Factor**	**Density**	**Other comments**
			**(g/g)**	**(g/event)**	**(events / day)**	**(g/g)**	**(-)**	**(hr)**	**(m^3/hr)**	**(m^3)**	**(cm^2)**	**(-)**	**(g/cm^3)**	
												transferred onto dermal wipe was 13.8 ug/m^2		
AC6: Leather articles	Footwear (shoes, boots)	TRA										1		
		RIVM toy fact sheet										leaching factor azo dyes from leather shoes, based upon unwashed items, ranged from 0.149-0.39 g dye per g product		
AC8: Paper articles	Printed paper (papers, magazines, books)	TRA										1.00		
	Printed paper (papers, magazines, books)	Spaan											newspaper 0.61 - 0.69; books, magazines,	

	Relevant Route:		I, D,O ,	I	I,D, O	I	I	I	I	I	D	D		
Descriptor	Product Subcategory	Reference	Product Ingredient fraction	Amount Product Used per	Frequency of Use	Fraction Released to Air	Dilution Fraction	Exposure Time	Inhalation Rate	Room Volume	Contact Area	Transfer Factor	Density	Other comments
			(g/g)	(g/event)	(events / day)	(g/g)	(-)	(hr)	(m^3/hr)	(m^3)	(cm^2)	(-)	(g/cm^3)	
													catalogues (non glossy) 0.72; magazines, catalogues (glossy) 1.11 - 1.16; home printing document fine paper 0.78	
	Printed paper (papers, magazines, books)	Spaan										transfer factors median to 75th ptile range for ink component: spiked plates 0.28-0.45%, printed paper: as % of full article thickness 0.0029 - 0.0065%, as upper 10 um of article 0.22-0.49%		

	Relevant Route:		I, D,O ,	I	I,D, O	I	I	I	I	I	D	D		
Descriptor	Product Subcategory	Reference	Product Ingredient fraction	Amount Product Used per	Frequency of Use	Fraction Released to Air	Dilution Fraction	Exposure Time	Inhalation Rate	Room Volume	Contact Area	Transfer Factor	Density	Other comments
			(g/g)	(g/event)	(events / day)	(g/g)	(-)	(hr)	(m^3/hr)	(m^3)	(cm^2)	(-)	(g/cm^3)	
AC10: Rubber articles	Footwear (shoes, boots)	TRA	0.1	800	1	1	0.17	8.0	1.37	20		1.00		
	Shoes made from rubber or plastic	Schneider		75th pctile: 190 (95th 302) weight of shoe	75th pctile: 0.4 (95th 0.9)			75th pctile: 3 (95th 9)						
AC11: Wood articles	Furniture (chair)	TRA	0.1	1300	1	1	0.29	4.0	1.37	20		1.00		
		RIVM toy fact sheet										dislodgeable formulation of metals from preserved wood 0.00012 - 0.688 ug/cm^2		
		RIVM toy fact sheet										transfer coefficient of 1040 cm^2/hr for fingers and 15940 cm^2/hr for whole body for children 2-5 years		

	Relevant Route:		I, D,O,	I	I,D, O	I	I	I	I	I	D	D		
**Descriptor**	**Product Subcategory**	**Reference**	**Product Ingredient fraction**	**Amount Product Used per**	**Frequency of Use**	**Fraction Released to Air**	**Dilution Fraction**	**Exposure Time**	**Inhalation Rate**	**Room Volume**	**Contact Area**	**Transfer Factor**	**Density**	**Other comments**
			**(g/g)**	**(g/event)**	**(events / day)**	**(g/g)**	**(-)**	**(hr)**	**(m^3/hr)**	**(m^3)**	**(cm^2)**	**(-)**	**(g/cm^3)**	
	Walls and flooring (also applicable to non-wood materials)	TRA	0.5	3000	1	1	0.17	8.0	1.37	20		1.00		
AC13: Plastic articles	Plastic, larger articles (plastic chair, PVC-flooring, lawn mower, PC)	TRA	0.43	8000	1	1	0.17	8.0	1.37	20		1.00		
		Spaan											PVC flooring 0.95 - 1.8 g/cm^3, thickness 0.18 - 0.5 cm (typical 0.2 cm, specialty products up to 0.4 cm)	

	Relevant Route:		I, D,O ,	I	I,D, O	I	I	I	I	I	D	D		
**Descriptor**	**Product Subcategory**	**Reference**	**Product Ingredient fraction**	**Amount Product Used per**	**Frequency of Use**	**Fraction Released to Air**	**Dilution Fraction**	**Exposure Time**	**Inhalation Rate**	**Room Volume**	**Contact Area**	**Transfer Factor**	**Density**	**Other comments**
			**(g/g)**	**(g/event)**	**(events / day)**	**(g/g)**	**(-)**	**(hr)**	**(m^3/hr)**	**(m^3)**	**(cm^2)**	**(-)**	**(g/cm^3)**	
		Spaan										transfer factors median to 75th ptile range DEHP, DINCH: spiked plates 0.0.24-1.08% , PVC flooring DEHP, DnBP, DiBP, DINCH:as % of full article thickness 0.00007-0.004%, as upper 10 um of article 0.04-1.5%		
	Plastic, small articles (ball pen, mobile phone)	TRA	0.43	75	1	1	0.17	8.0	1.37	20		1.00		
	Ball point pen/pencil	Schneider		75th pctile: 15 (95th 30) weight of writing utensil	75th pctile: 0.9 (95th 0.9)			75th pctile: 1 (95th 4)						Self reported duration of pen touching mouth was 0.1 - 150 min/day but time is not considered in oral algorithm

References

Bremmer HJ, van Veen MP. Children’s toys fact sheet: to assess the risks for the consumer. RIVM Report no 612810012/2002. RIVM, Bilthoven 2002.

Gorman Ng M, Semple S, Cherrie JW, Christopher Y, Northage C, Tielemans E et al. The relationship between inadvertent ingestion and dermal exposure pathways: A new integrated conceptual model and a database of dermal and oral transfer efficiencies. Ann Occup Hyg 2012; 56(9): 1000–1012.

Quadros ME, Pierson IV R, Tulve NS, Willis R, Rogers K, Thomas TA, et al. Release of silver nanotechnology-based consumer products for children. Env Sci Technology 2013; 47:8894-8901.

Schneider K, Recke S, Kaiser E, Götte S, Berkefeld H, Lässig J, et al. Consumer behaviour survey for assessing exposure from consumer products: a feasibility study. J Exposure Science and Environmental Epidemiology. 2019; 29(1): 83-94.

Spaan S, de Brouwere K, Geerts L, Marquart H. DRESS Guidance document for assessment of dermal exposure of consumers to substances in articles: suggestions for refinement of ECETOC TRA / improvements for the dermal exposure assessment strategy. 2014, Report no. 4.

SI10. Benchmarking TRA AC Predictions with Modeled or Measured Data

Limited data were available for benchmarking exposures via articles.

For inhalation exposures, several measurements of total Volatile Organic Carbon Compound emissions were available for electronics, and these can be used to benchmark inhalation estimates associated for those categories (Table SI-10.1 provides the data, associated exposures are found in main text Table 3).

Table SI-10.1 Malgrem et al., 2003 - Chamber emissions measurements				
		New sets 1999	after 4 months aging	Comments
		µg/set-hour	µg/set-hour	
TV Sets	TVOC	189-2036	<25 – 157	Measured VOCs and SVOCs in chambers; authors indicate air concentrations typically reach a maximum after about 6 hours of use then decline for VOCs, for SVOCs slowly increase over time reaching equilibrium at 100 hours or longer.
TV Sets	individual chemicals	<0.00005 - 236	<0.00005-35	
		µg/monitor-hr		
Computer monitors	TVOC	36-11099		
Computer monitors	single largest emission	300		

For dermal exposures, several more modeling studies were available:

Spaan et al., 2014 provide 4 case studies using specific substances. TRA values are shown for all defaults, for weight fraction modified to the case study specifics, and then with other factors also adjusted to the case study conditions. Estimates were compared to predictions based upon a mass balance approach and diffusion based approach. In the mass balance approach, exposure was based upon the amount of substance present in the entire thickness of the article for either the skin surface area or for the contacted article surface area (the latter approach was used for flooring, which is significantly greater than the TRA default skin contact area). Numeric values were provided for both textile scenarios. For the printer paper and flooring studies, most values had to be estimated from results provided in figures. The equations used and specific values used were provided, and so were used for replicating the analysis. For the flooring case study, in one case the calculated value differed from the value estimated in the figure, both are provided here.

Figure SI-10.1a Spaan Textile Case Study – DMF in T-shirt

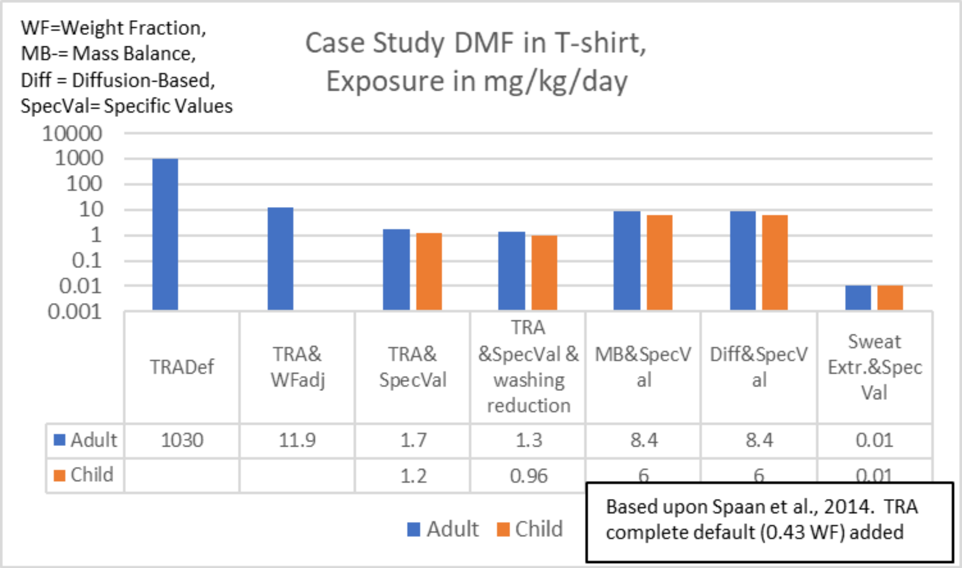

Figure SI-10.1b Spaan Textile Case Study – dioxin in contaminated T-shirt

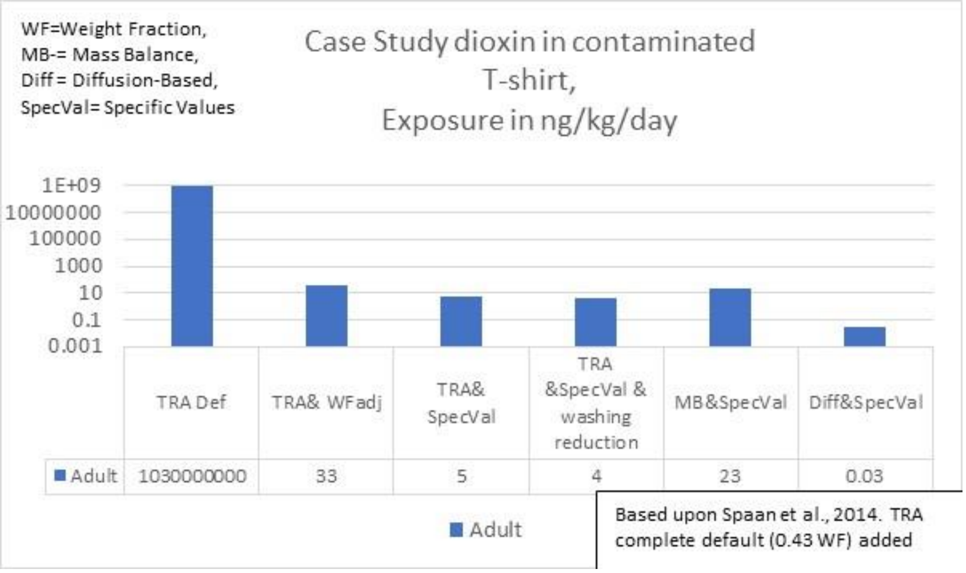

Figure SI -10.2 Spaan printed paper case study – DB360 in printed paper

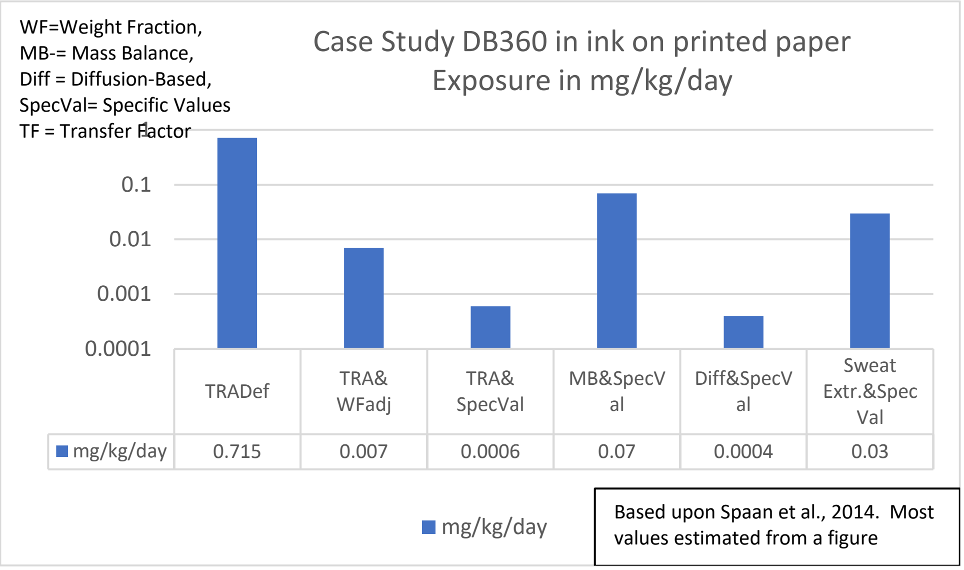

Figure SI-10.3 Spaan flooring case study – DEHP in PVC flooring

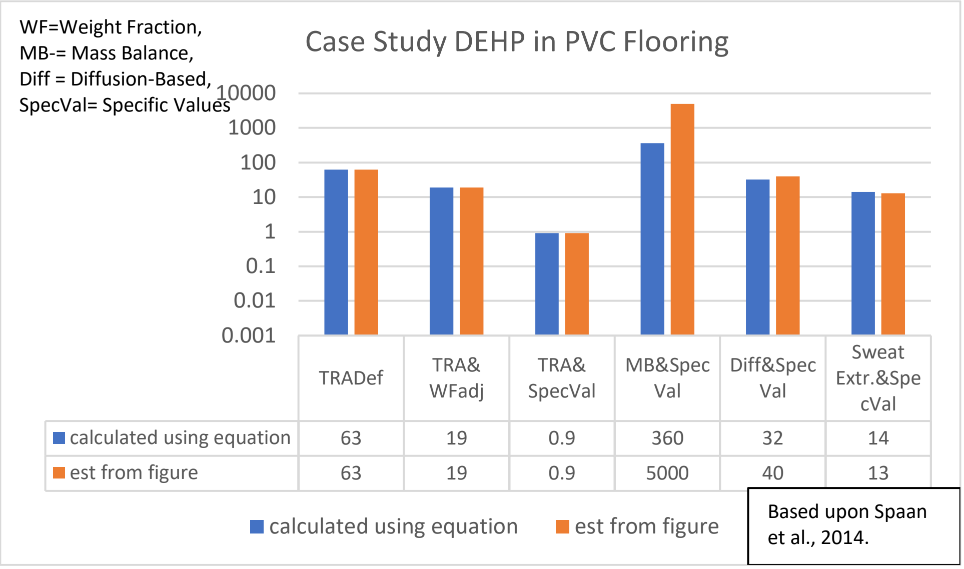

Delmaar et al., (2013) compared TRA predictions to those using a diffusion based approach. Here, emissions from the product during the exposure period are used, and are equated to

external exposure potential. Numeric values were not provided, but diffusion-based values were estimated from the figure in Delmaar (Table SI 10.2), TRA values were calculated. These values were then converted to mg/kg/day using a 60 kg body weight for inclusion in Figure 3 and discussion in Table 3 of the main text. The diffusivity predictions are about 2 orders of magnitude greater than the TRA.

Table SI-10.2 Estimated article emissions to dermis based upon Delmaar et al., 2013

	TRA-calculated	Diffusivity Layer-estimated from Delmaar et al., 2013 figure
AC5: Textiles- flooring	9	2000
AC5: Textiles- bedding	1.5	95
AC5: Textiles- clothing	62	800
AC10: Rubber articles - flooring	0.2	20
AC10: Rubber articles - foot wear	0.2	60
AC10: Rubber - handles	0.04	3
AC11: Wood -flooring	0.2	30
AC11: Wood – furniture	0.9	95
AC13: Plastic - flooring	3.8	300
AC13: Plastic - small articles	0.2	0.2

Several additional studies or estimates developed are provided individually in the following SI sections and/or in the main text.

References:

Delmaar JE, Bokkers BGH, ter Burg W, van Engelen JGM. First tier modeling of consumer dermal exposure to substances in consumer articles under REACH: A quantitative evaluation of the ECETOC TRA for consumers tool. Reg Tox Pharm 2013; 65:79-86.

Malmgren-Hansen B, Olesen S, Pommer K, Winther Funch L, Pedersen E, Willum O, et al. Danish Technological Institute & Technical University of Denmark. Survey of chemical substances in consumer products, Survey no. 32-2003: Emission and evaluation of chemical substances from selected electrical and electronic products. 2003.

Spaan S, de Brouwere K, Geerts L, Marquart H. DRESS Guidance document for assessment of dermal exposure of consumers to substances in articles: suggestions for refinement of ECETOC TRA / improvements for the dermal exposure assessment strategy. 2014, Report no. 4.

SI11 Benchmarking TRA Dermal Predictions with USEPA 2012 Approach

To benchmark the TRA calculations, USEPA guidance for estimating exposures from contact with materials impregnated with pesticides was used (USEPA 2012). Exposures were calculated as:

$$E = WF * MD * TE * SA$$

Where:

WF= weight fraction of substance in treated material (g/g)

MD= Material weight:surface area density (mg material/cm^2)

TE = Daily material-to-skin transfer efficiency (fraction/day)

SA = Skin contact Area

The weight per surface area by material type is summarized in Table SI11.1. Daily skin transfer efficiencies are 0.06/day for textiles or carpeting and 0.08/day for flooring on hard surfaces. These default values were developed by USEPA based upon the analysis of Beamer et al 2009 and the Non-Dietary Task Force. Using the TRA default product ingredient weight fraction, exposed skin surface area and body weight with the material weight per surface area and daily skin transfer efficiency yields the following exposure estimate for carpets:

Exposure = 120 mg/cm^2 X 0.1 X 0.06 X 8750 cm^2 = 6300 mg; for a 60 kg body weight = 105 mg/kg/day

In comparison the TRA estimate is 8750 mg/day or 146 mg/kg/day. While this approach has limitations, it is expected to yield conservative dermal exposures for materials evaluated in the EPA document. This is because the default material-to-skin transfer efficiency rates are based on data from carpets and hard surfaces that have had a chemical applied to their external surface rather than incorporated into the article matrix. If the higher daily skin transfer efficiency value of 0.08 was applied to the diffusion layer model estimates of Delmaar et al. (2013), the results would be similar to or an order of magnitude greater than TRA predictions. We observed that the application of a daily transfer efficiency has limitations as in reality the value will depend upon the amount present in or on the article, the nature of the article and of the skin contact, and in this case is a relative value expressed as a fraction of total.

Table SI-11.1 USEPA Recommended weight-to-surface area values for various types of fabrics and materials. (Basis USEPA 2012 Table 9-1).	
Material	Material Weight: Surface Area (mg/cm^2)
Textile: Cotton	20
Textile: Light Cotton/Synthetic Mix	10
Textile: Heavy Cotton / Synthetic Mix	24
Textile: All Synthetics	1
Household Carpets	120
Plastic Polymers	100
Vinyl Flooring	390

References:

Delmaar JE, Bokkers BGH, ter Burg W, van Engelen JGM. First tier modeling of consumer dermal exposure to substances in consumer articles under REACH: A quantitative evaluation of the ECETOC TRA for consumers tool. Reg Tox Pharm 2013; 65:79-86.

Beamer, P; Canales, RA; Leckie, JO. (2009). Developing probability distributions for transfer efficiencies for dermal exposure. Journal of Exposure Science and Environmental Epidemiology. 19: 274-283.

USEPA Standard Operating Procedures for Residential Pesticide Exposure Assessment. USEPA Health Effects Division, Office of Pesticide Programs, Office of Chemical Safety and Pollution Prevention, Washington DC. 2012

SI12 Comparison of TRA Consumer v.3.1 dermal exposure predictions with an ex vivo dermal migration and permeation data for BaP from Bartsch et al. (2016)

The paper of Bartsch et al. (2016) presents data on the migration of PAHs from consumer products into aqueous sweat simulant, aqueous ethanol solution, as well as the data on its migration and penetration into human skin. Product specimens were either submerged in simulant, or placed directly on test skin samples in Franz cell chambers to simulate dermal contact.

The rationale behind the study design with Franz cells was that real exposure conditions cannot adequately be simulated by applying dissolved chemicals onto test skin in vitro because of potential vehicle effect impacting dermal absorption. For example, several studies pointing to significant differences in percutaneous absorption of B[a]P when applied as contaminant in soil compared to B[a]P dissolved in acetone.

By using real commodity goods brought into direct contact with human skin, a penetration model has been developed by Bartsch et al. to closely mirror everyday dermal exposure scenarios. The article specimen used were contaminated with B[a]P in the range of 57–266mg/kg material.

The results of the Franz cell chamber assay using human skin ex vivo show that a total of 102 ng/cm2 B[a]P penetrated the skin when brought into contact and incubated for 24h with the material of a conventional hammer handle. It should be noted that the thickness of the specimen article was not specified in the paper; could be assumed 2 mm given in Delmaar et al. (2013) for rubber handles. Alternatively, it can be calculated based on the OECD TGD 428 on dermal in vitro absorption recommendation for maximum dosing of solid substances (i.e. 0.005cm; see above) (OECD, 2004).

Figure SI-12.1 illustrates dermal exposures estimated with the TRA TL-based model, the Delmaar et al. (2013) simplified diffusion-based layer model (mimicking both TRA scenario and Bartsch et al. scenario) and the data from ex vivo experiments (one sample).

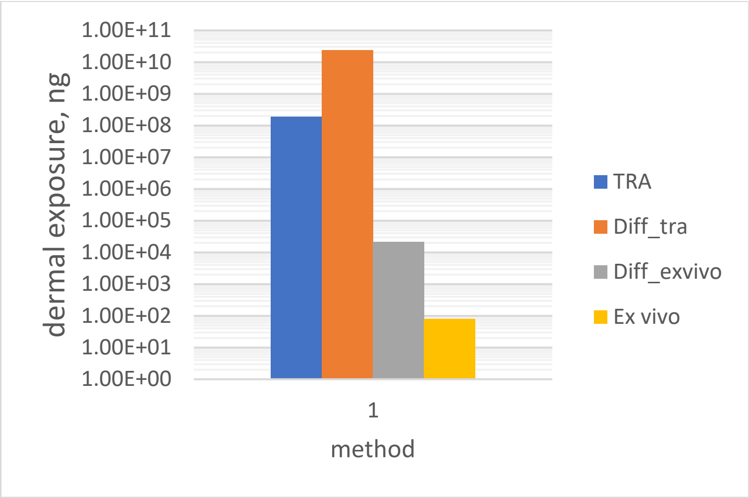

Figure SI-12.1. Dermal exposure to BaP upon direct skin contact with hammer handle (TRA AC13 Plastic, small articles) estimated using different approaches.

The table below provides the default values for input parameters used in each approach.

Table SI-12.1. Basis for analysis					
**Parameter**	**Unit**	**Model**			
		**ECETOC TRA Consumer v3.1**	**RIVM diffusion model**		**ex vivo Bartsch et al., 2016 (sample 2)**
			**TRA settings**	**ex vivo settings**	
article matrix		plastic	PE	PE	chlorosulfonated PE
D of BaP in article matrix	m^2/s	-	1.00E-11	1.00E-11	-
article thickness	m	-	0.002	0.002	0.002
layer thickness in contact with skin	m	0.00001	-	-	-
density	g/m^3	1000000	1000000	1260000	1260000
substance fraction	g/g	0.43	0.43	0.000166	0.000166

Table SI-12.1. Basis for analysis					
contact area	m^2	0.04288	0.04288	0.0000785	0.0000785
contact time	s	-	86400	86400	86400
**A0 / total BaP mass in the tested sample**	g	-	3.69E+01	3.28E-05	3.28E-05
total dermal exposure	ng	1.84E+08	2.42E+10	2.16E+04	8.01E+01
**total dermal exposure**	**g**	**1.84E-01**	**2.42E+01**	**2.16E-05**	**8.01E-08**

Bartsch et al. confirmed PAH accumulation in human epidermis by visualization with fluorescence signal. B[a]P dissolved in acetonitrile was applied as positive control (Figure 4A). Direct contact with sample #3 (266 mg/kg B[a]P) and human skin ex vivo resulted in an accumulation of PAHs in the upper epidermal layer (Figure 4B), but not in subepidermal layers. It can be also seen that the BaP layer formed on skin surface is not uniform and that its thickness is clearly <100um (0.01cm).

References

Bartsch N., Heidler, B., Hutzler C., Luch A. Skin preparation of PAHs: a solvent-based in vitro approach to assess dermal exposures against benzo[a]pyrene and dibenzopyrenes. J Occup Environ Hyg 2016; 12:969-979.

Delmaar JE, Bokkers BGH, ter Burg W, van Engelen JGM. First tier modeling of consumer dermal exposure to substances in consumer articles under REACH: A quantitative evaluation of the ECETOC TRA for consumers tool. Reg Tox Pharm 2013; 65:79-86.

Delmaar JE, Bokkers BGH, ter Burg W, van Engelen JGM. First tier modeling of consumer dermal exposure to substances in consumer articles under REACH: A quantitative evaluation of the ECETOC TRA for consumers tool. Reg Tox Pharm 2013; 65:79-86.

OECD (2004), Test No. 428: Skin Absorption: In Vitro Method, OECD Guidelines for the Testing of Chemicals, Section 4, OECD Publishing, Paris, https://doi.org/10.1787/9789264071087-en.
